# Recent Advances in the Development of Non-PIKKs Targeting Small Molecule Inhibitors of DNA Double-Strand Break Repair

**DOI:** 10.3389/fonc.2022.850883

**Published:** 2022-04-06

**Authors:** Jeremy M. Kelm, Amirreza Samarbakhsh, Athira Pillai, Pamela S. VanderVere-Carozza, Hariprasad Aruri, Deepti S. Pandey, Katherine S. Pawelczak, John J. Turchi, Navnath S. Gavande

**Affiliations:** ^1^Department of Pharmaceutical Sciences, Eugene Applebaum College of Pharmacy and Health Sciences, Wayne State University, Detroit, MI, United States; ^2^Department of Medicine, Indiana University School of Medicine, Indianapolis, IN, United States; ^3^NERx Biosciences, Indianapolis, IN, United States; ^4^Department of Biochemistry and Molecular Biology, Indiana University School of Medicine, Indianapolis, IN, United States; ^5^Molecular Therapeutics Program, Barbara Ann Karmanos Cancer Institute, Wayne State University School of Medicine, Detroit, MI, United States

**Keywords:** DNA repair and DNA damage response (DDR), DNA double-strand break (DSB) repair, non-PIKKs inhibitors, non-homologous end joining (NHEJ), homology directed repair (HDR), single-strand annealing (SSA), polymerase theta-mediated end joining (TMEJ), synthetic lethality

## Abstract

The vast majority of cancer patients receive DNA-damaging drugs or ionizing radiation (IR) during their course of treatment, yet the efficacy of these therapies is tempered by DNA repair and DNA damage response (DDR) pathways. Aberrations in DNA repair and the DDR are observed in many cancer subtypes and can promote *de novo* carcinogenesis, genomic instability, and ensuing resistance to current cancer therapy. Additionally, stalled or collapsed DNA replication forks present a unique challenge to the double-strand DNA break (DSB) repair system. Of the various inducible DNA lesions, DSBs are the most lethal and thus desirable in the setting of cancer treatment. In mammalian cells, DSBs are typically repaired by the error prone non-homologous end joining pathway (NHEJ) or the high-fidelity homology directed repair (HDR) pathway. Targeting DSB repair pathways using small molecular inhibitors offers a promising mechanism to synergize DNA-damaging drugs and IR while selective inhibition of the NHEJ pathway can induce synthetic lethality in HDR-deficient cancer subtypes. Selective inhibitors of the NHEJ pathway and alternative DSB-repair pathways may also see future use in precision genome editing to direct repair of resulting DSBs created by the HDR pathway. In this review, we highlight the recent advances in the development of inhibitors of the non-phosphatidylinositol 3-kinase-related kinases (non-PIKKs) members of the NHEJ, HDR and minor backup SSA and alt-NHEJ DSB-repair pathways. The inhibitors described within this review target the non-PIKKs mediators of DSB repair including Ku70/80, Artemis, DNA Ligase IV, XRCC4, MRN complex, RPA, RAD51, RAD52, ERCC1-XPF, helicases, and DNA polymerase θ. While the DDR PIKKs remain intensely pursued as therapeutic targets, small molecule inhibition of non-PIKKs represents an emerging opportunity in drug discovery that offers considerable potential to impact cancer treatment.

## Introduction

### DSB Repair Pathways

**Figure 1:**
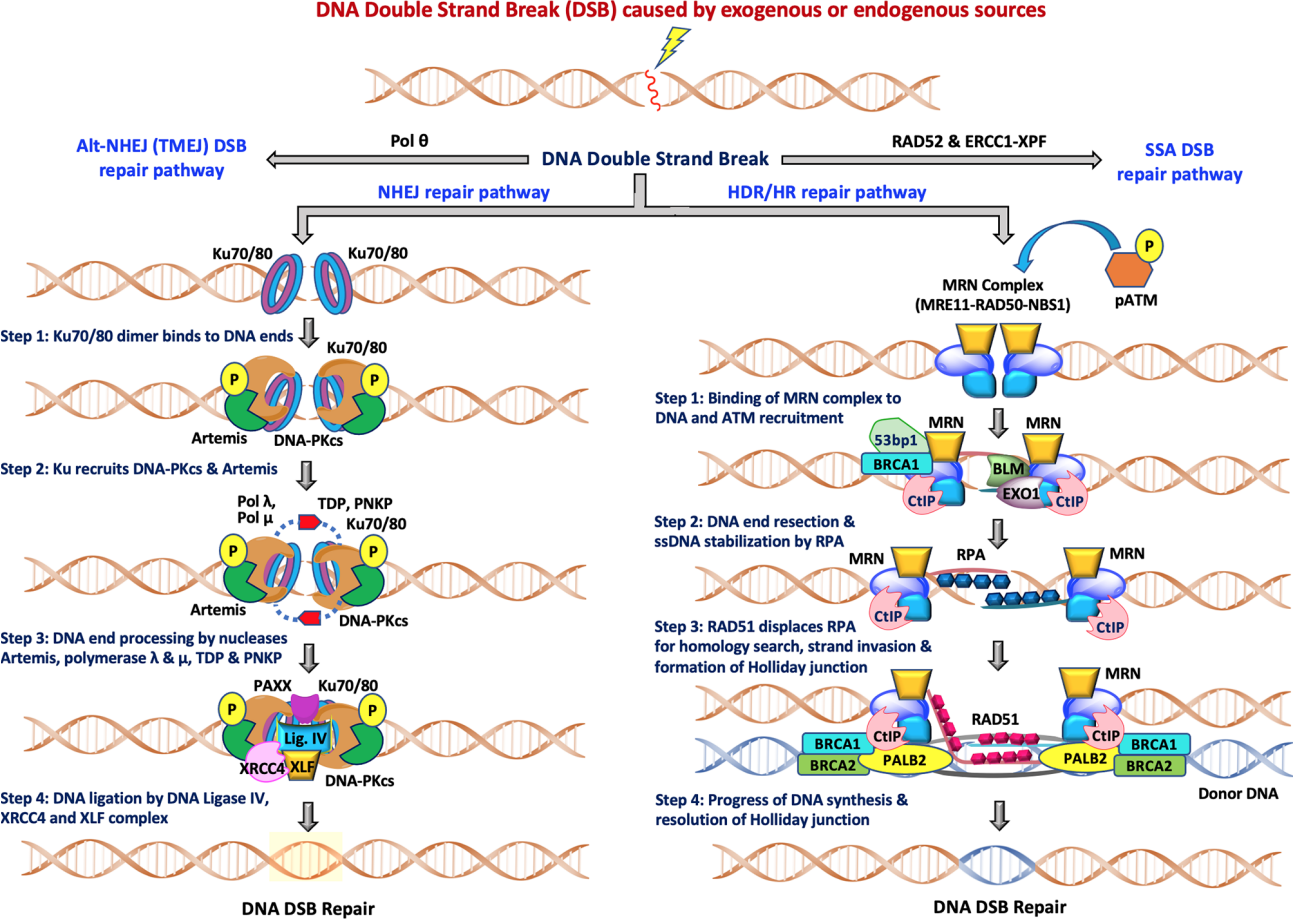
The two major pathways of DNA double-strand break repair: During NHEJ, the DNA double strand sites are initially recognized by heterodimeric Ku70/80. This is followed by recruitment of DNA-PKcs and Artemis, DNA end processing by Artemis, polymerase λ and μ, TDP and PNKP and finally ligation of DSB breaks by Ligase IV/XRCC4/XLF complex for completion of the repair pathway. The other accessory proteins like APLF, PAXX and XLF also participate in the repair functions. During HDR/HR, DSBs are recognized and resected by the MRN complex to generate a 3’ overhang. BRCA2/RAD51, along with other RAD51 paralogs, binds to the RPA coated ssDNA tails after which RAD51 replaces RPA in a BRCA1-and BRCA2-dependent process, forming a presynaptic filament. Upon strand invasion, D-loop formation and DNA repair synthesis can be resolved through Holliday junction, after which distinct independent pathways can operate to complete the HDR repair pathway. NHEJ is available throughout interphase while HDR is restricted to S/G2 phases of the cell cycle.

DNA double-strand breaks (DSB)s are considered the most lethal of all DNA lesions. DSBs may be induced by various exogenous and endogenous factors, such as ionizing or ultraviolet radiation, genotoxic chemicals/chemotherapeutic agents, replication errors or collapsed replication forks, reactive oxygen species (ROS), free radicals, V(D)J recombination and abortive enzymatic activity (1–4). Unrepaired DSBs can lead to cell death, as persistent DSBs can trigger apoptosis (5–7). Moreover, misrepair or inaccurate repair of DSBs can lead to pathological genomic alterations resulting in senescence, loss of heterozygosity or chromosomal translocations which can ultimately result in oncogenesis (8). Interestingly, DSBs are routinely generated in the process of V(D)J recombination in naïve B- and T-lymphocytes to generate a diverse array of immunoglobulins and T-cell receptors, and the role of DSB repair in these processes has recently been reviewed (9). Aside from posing risk for cancer, DSBs are implicated in premature aging, and DSB repair capacity generally declines with age (10). In mammalian cells, the majority of DSBs are repaired *via* non-homologous end joining (NHEJ) or the homology directed repair (HDR)/homologous recombination (HR) pathways (**Figure 1**). In addition to NHEJ and HDR, less frequently involved or backup pathways including single-strand annealing (SSA) and alternative NHEJ (alt-NHEJ) also contribute to DSB repair (11–13). Alt-NHEJ is also called microhomology-mediated end joining (MMEJ) and more recently referred to as polymerase theta-mediated end joining (TMEJ) as recently reviewed by Ramsden et al. (14). Canonical or classical NHEJ is the predominant pathway in human cells and is active throughout the cell cycle, rapidly repairing up to ∼80% of all DSBs (15, 16). HDR is a much slower DSB repair process and is restricted exclusively to S and G2 phases of the cell cycle due to the requirement for a homologous DNA sequence or availability of sister chromatid as a template for the repair process (3).

**Figure 1** (left) depicts the various steps in the NHEJ repair pathway, and these can be summarized into four specific steps which include: (i) DNA termini recognition by Ku70/80; (ii) bridging of the two DNA ends also known as formation of the synaptic complex; (iii) DNA end processing, and finally (iv) DNA ligation (16–20). Following the induction of DSB by exogenous sources like ionizing radiation (IR) or chemotherapeutics, the NHEJ pathway is initiated by the binding of the heterodimeric Ku70/80 to the end of DNA break which recruits DNA-dependent protein kinase catalytic subunits (DNA-PKcs) to form the DNA-PK holoenzyme (DNA-PK). The DNA-PKcs serine/threonine protein kinase activity is activated once bound to a DNA terminus in the presence of Ku70/80. The formation of the DNA-PK complex stabilizes the two DNA ends at the site of the break by forming a synaptic complex that holds the two DNA termini together (21, 22). DNA-PK catalyzes both autophosphorylation and phosphorylation of other target proteins including Ku70/80, Artemis, polynucleotide kinase 3′-phosphatase (PNKP) and XRCC4. When required, DNA end processing relies on the kinase activity of DNA-PKcs, endonuclease cleavage activity of Artemis, nucleotide addition and modification by DNA polymerases (Pol X family polymerases such as pol λ and pol μ), tyrosyl-DNA phosphodiesterase (TDP), and PNKP. Finally, the DNA Ligase IV/XRCC4/XLF complex is recruited to DNA termini and catalyzes ligation of the DNA DSB.

The HDR pathway is depicted in stepwise fashion in **Figure 1** (right) and can be summarized as: (i) binding of the MRN complex to each of the damaged dsDNA ends; (ii) end resection by the MRN complex, CtIP, EXO1, BLM and stabilization of the ssDNA overhangs by RPA binding; (iii) RAD51 displacement of RPA and formation of the Holliday junction with a homologous sequence; and (iv) resolution of the Holliday junction. The MRN complex (MRE11-RAD50-NBS1) is crucial for recognition of homologous sequences, performing end resections to generate ssDNA tails and nucleofilament formation by Replication Protein A (RPA) which is eventually replaced by RAD51. BRCA1 and BRCA2 also facilitate RAD51 filament nucleation. After recruitment of RAD51, a homology search can now be performed that when successful allows invasion of the non-resected strand into the homologous template and resulting D-loop formation of the displaced template strand. Capture of the D-loop by the broken dsDNA produces a Holliday junction that is later resolved by endonuclease activity, completing HDR. The distinct independent pathways that can operate to complete the HDR repair pathway are reviewed elsewhere (23, 24). Decades of investigation have established the importance of NHEJ, HDR, TMEJ and SSA pathways, the roles of the various factors/proteins involved in these pathways and how these factors coordinate and regulate distinct steps of these pathways at the molecular level. More detailed descriptions of these pathways can be found in recent reviews (11, 14, 16, 25, 26).

It is worth mentioning that a prerequisite to repair of DSBs is that the lesion is accessible which typically requires histone modifications and reorganization of chromatin (27). Acetylation of histones promotes DNA unraveling by electronegative repulsion which enables the DNA repair machinery access to the DSB. Targeting histone deacetylases (HDACs) with small molecule drugs or through promoting their degradation by inhibition of the deubiquitinase conferring HDAC stability are other strategies to enhance radiosensitivity (28, 29). To complicate matters further, the chromatin state must be returned to the pre-existing state after DSB repair.

Telomeres are repetitive DNA elements that protect chromosomal termini and prevent their false recognition as DSBs which could activate a deleterious DDR such as NHEJ-mediated chromosomal fusion or cyclization (30, 31). DDR activation and maintenance at telomeres depends on the biogenesis and functions of the site-specific small non-coding RNAs, also known as DNA damage response RNAs (DDRNAs) (32). Telomeres shorten with cell division during replicative senescence (due to the end-replication problem). Excessive telomeric erosion has been shown to contribute to a persistent DDR and have been implicated in the ageing process and disease development alongside with a host of lifestyle factors, stresses, and environmental exposures (33). The maintenance of telomere homeostasis is critical for chromosome stability in proliferating cancer cells which usually have higher telomerase activity compared to normal cells (34). In general, cancer cells maintain telomeres at shorter lengths compared to normal cells. Besides preventing chromosome shortening, telomerase also intervenes to thwart the DSB response through protein-protein interactions with specialized telomere-binding proteins. There are several proteins involved in the DSB response which are also localized to telomeres and participate in telomere homeostasis (34). For example, the Ku protein has been demonstrated to be localized to telomeres and serves to protect the telomere against fusions. Particularly, depletion of the Ku heterodimer leads to severe telomere erosion and loss of cell viability (35, 36). Overall, telomerase has been an attractive target for the development of effective cancer therapeutics as it has shown overexpression in the majority of human cancers. The anti-telomerase therapeutics can provide selective destruction of cancer cells while noncancerous cells are predominantly spared owing to telomerase silencing in most normal somatic cells (37).

RecQ and MCM (Minichromosome Maintenance) helicases, a family of DNA unwinding enzymes, play important roles in genomic stability through diverse roles in DNA recombination, replication and repair (38, 39). RecQ proteins can function both at early and late stages during repair of DSB. In addition, RecQ helicase proteins BLM (Bloom syndrome) and WRN (Werner syndrome) are also involved in telomere homeostasis as well as the processing and re-initiation of stalled replication forks (40, 41). The CMG helicase complex composed of three replication factors (Cdc45/Mcm2-7/GINS) is required to unwind dsDNA to generate the ssDNA template during DNA replication (42). A stalled replication forks by MCM helicases can lead to a DSB as well as chromosomal rearrangements, which can eventually recruit RecQ proteins for repair due to their functional connections (39, 43). Targeting these helicases has immense importance in developing new therapeutics against various cancers.

The recent advances in the field of the mitochondrial DNA damage response (mtDDR) warrant consideration of the nonnuclear genome in the development of inhibitors of non-PIKKs in DSB repair, as several non-PIKKs are now implicated in the mtDDR (44–46). In humans, mitochondria contain a polyploid genome comprised of a heterogenous mixture of ~16.5 kbp circular DNA chromosomes. Consistent with endosymbiotic ancestry from proteobacteria, the mitochondrial DNA (mtDNA) replication, transcription, and DDR machinery includes gene products evolutionary derived from eukarya, bacteria, and T7-like bacteriophages (47). mtDNA replication, transcription, and damage repair occur independently of their nuclear counterparts. The maternally inherited mitochondrial genome includes 37 genes encoding all required mitochondrial tRNAs and rRNAs and 13 core proteins of complexes I, III, IV, and V of the electron transport chain (ETC).

mtDNA is subjected to damage by the same sources as nuclear DNA, although the proximity of mtDNA to the ETC complexes heightens the risk for ROS-induced DNA damage. Aberrations of mtDNA including mutations or deletions are associated with the development of diseases including Kearns-Sayre syndrome, Pearson syndrome, cancer, aging, Alzheimer’s disease, and diabetes among others (44–46). Unlike the nucleus, there appears to be no role for classical NHEJ in the mitochondria where DSBs are predominantly repaired by the alt-NHEJ pathway. Mitochondrial alt-NHEJ proceeds independent of Ku70/80 and is dependent on Ligase III and MRE11 among others (48). DNA polymerase θ also appears to play a role in mitochondrial alt-NHEJ but in an error-prone manner unlike in the nucleus where fidelity is high (49, 50). Besides alt-NHEJ there is now mounting evidence to suggest that HDR may also repair DSBs in mtDNA, although possibly with nuances and a requirement for additional proteins. Four-way junctions and HDR mediators RAD51, RAD51C, XRCC3, and MRE11 have been detected in the mitochondria, and functional assays have demonstrated DSB repair in mitochondria consistent with HDR (51–54). Unrepaired mtDNA may be compensated for by undamaged mitochondrial chromosomes or trigger mitochondrial translesion synthesis, fusion, fission, or mitophagy to manage or purge the damaged mtDNA (44). There is evidence to suggest that mtDNA damage is sufficient to induce apoptosis or enhanced immunogenicity independent of nuclear DNA damage (55–58). Given the roles of non-PIKKs in the mtDDR, inhibitors should be assessed for their effects on mitochondrial HDR and alt-NHEJ. In similar fashion, the proapoptotic and immunogenic effects seen with targeted mtDNA damage highlights a potential for mitochondrial-selective anticancer drugs.

The innate immune response to cancer is favored by heightened DNA damage such as by unrepaired DSBs, although the adaptive immune response effectors B cells and T cells require intact DSB repair to repair the DSBs they routinely generate in V(D)J recombination. Accordingly, the strategy for targeting DSB repair in cancer will need to balance these opposing effects on the immune system. A precondition to repairing DSBs is access to the lesion by the DDR machinery through chromatin modification, and inhibition of HDACs may provide a way to block DSB repair upstream of DDR effector scaffolding at the damaged site.

An emerging area of research within the field of DNA damage and repair is the role of non-coding RNAs (ncRNAs) in the DDR to DSBs which has recently been reviewed (59–61). ncRNAs are classified as being either long ncRNAs (lncRNAs) or short ncRNAs (sncRNAs) depending on whether length exceeds 200 nucleotides. A subclass of lncRNAs is damage-induced lncRNAs (dilncRNAs), which are transcribed bidirectionally from DSBs after the arrival of the MRN complex and promote HDR by localizing RAD51, BRCA1, and BRCA2 to the lesion (59, 60). Micro RNAs (miRNAs), a subclass of sncRNAs, are typically derived from RNases such as DICER or Drosha and in the DDR regulate gene expression post-transcriptionally to select the DSB repair pathway employed, induce cell cycle arrest, and promote apoptosis where indicated (61). More broadly, ncRNAs are thought to serve as an alert to the presence of DSBs, to recruit DDR effectors to the lesion, and to temporarily bridge the broken ends in proximity, among other functions (59, 60). However, an improved understanding of ncRNAs in DSB repairs may produce additional opportunities for RNA-targeted therapeutic intervention. Overall, the multifunctional role of DNA repair and DDR pathways increases the complexity and difficulty of targeting DNA repair pathways for a positive clinical outcome.

### Biological Impacts of DSB Repair in Cancer

DNA repair pathways play a central role in protecting cells against genomic instability and mutations. Moreover, DNA repair pathways play a multifaceted role in cancer onset, progression, metastasis, and ultimately on clinical outcome of cancer therapeutic strategies. Aberrations of DNA repair proteins or genes can predispose the cells to carcinogenesis and this vulnerability can be therapeutically exploited to preferentially kill tumor cells. The relative functionalities of the DNA repair and DNA damage response (DDR) pathways, whether defective, deficient, or hyperactive, as well as the ability of cancer therapeutics to inhibit or activate DNA repair, all can influence a patient’s response to therapy (5, 62, 63). The upregulated DNA repair and DDR activity can promote disease progression and make cancer cells resistant to the treatment or cause post-treatment relapse.

Many well-known anticancer chemotherapeutics and IR impart their clinical efficacy by inducing DNA damage. DNA damaging agents such as etoposide, bleomycin, doxorubicin and IR (radiotherapy) exert their therapeutic efficacy by inducing DNA DSBs. Approximately 50% of all cancer patients worldwide with common epithelial malignancies (including lung, prostate, breast, colon, head and neck, and esophageal cancers) are subjected to radiation therapy as a component of their treatment regimen. Radiotherapy is very cost effective and in combination with other medical treatments has contributed to improved long-term survival in subsets of cancer patients. Despite advanced technical improvements and the fact that radiotherapy is one of the most effective forms of cancer treatment, many patients still suffer from detrimental locally recurrent disease or long-term chronic side effects after radiotherapy due to being treated with higher doses of radiation (64–66). Most importantly, radiotherapy and DNA damaging chemotherapeutics often lead to poor clinical response due to the development of intrinsic or extrinsic resistance. There are multiple factors involved in IR and drug resistance, among them increased capacity of DNA DSB repair is one of the major primary concerns, and in many cases, resistance to therapy is an adaptive response linked to hyperactive DSB repair mechanisms (64, 67). The overexpression or loss of function due to polymorphisms, mutations of core and processing NHEJ proteins such as Ku70/80, DNA-PKcs, Ligase IV/XRCC4, and HDR proteins such as MRN, BRCA1/2 and RAD51 have been implicated in reduced therapeutic efficacy of IR and DNA damaging chemotherapeutics. Research within cancer genomics, proteomics, and metabolomics has led to a deeper understanding of the molecular mechanisms driving the development of resistance. In response to DNA damage, the affected cells recruit functional proteins to initiate the DSB repair pathway that enhances the DNA lesion repair which ultimately leads to drug resistance.

The targeted inhibition of repair pathways is a novel and effective strategy to induce persistent DSBs and increase apoptosis of cancer cells. This strategy is particularly promising in the setting of combination therapy with DSB-inducing treatments such as radiotherapy or radiomimetic drugs or in combination with other DNA damaging drugs. However, where unrepaired DSBs fail to directly induce cell death, induction of the innate immune response may ensue. The interplay of the DDR and the innate immune response has recently been reviewed (9). Cytosolic DNA arising from damaged nuclear or mitochondrial DNA is recognized as a pathogen- or damage-associated molecular pattern (PAMP/DAMP) and ultimately induces stimulator of interferon genes (STING)-dependent signaling. The resulting production of interferons enhances the cellular antitumor response by the immune system. Intriguingly, several of the non-PIKKs targeted by ligands reviewed here such as MRN complex, DNA ligase IV, and XRCC4 appear to have dual roles in stimulating the innate immune response aside from their classical roles in DSB repair.

As cancer cells frequently harbor defect in genes of a DNA repair pathway, they may be increasingly reliant on the remaining available pathways to repair DNA damage occurring endogenously or in response to treatment. Defects in DNA repair in cancer cells thus presents a vulnerability to exploit synthetical lethal interactions where noncancerous cells would remain resilient. In cancer treatment, this has been typified using PARP inhibitors in cancers that are HDR-deficient. The synthetic lethality approaches have provided novel mechanisms to specifically target cancer cells while noncancerous cells can tolerate or repair the damage which is anticipated to reduce toxicity associated with treatment. The availability of DNA repair inhibitors targeting a variety of DSB repair and DDR mediators will allow the strategy of synthetic lethal interactions to be more broadly applied clinically and with greater efficacy.

In this review, we focus specifically on recent advances in the development of non-PIKKs (PI3 kinase-like kinases) DSB repair targeted inhibitors that can be exploited for effective chemo- or radio-sensitization and to enhance the efficiency of precise genome editing as well. PIKKs such as ATM, ATR, and DNA-PK which are involved in DNA repair and DDR, have received considerable attention recently as pharmacological targets, and several inhibitors have risen to clinical trials. The progress pertaining to the development of these inhibitors is reviewed elsewhere (62, 68–70).

## Recent Advances in the Development of Non-Pikks DSB Repair Inhibitors

**Figure 2:**
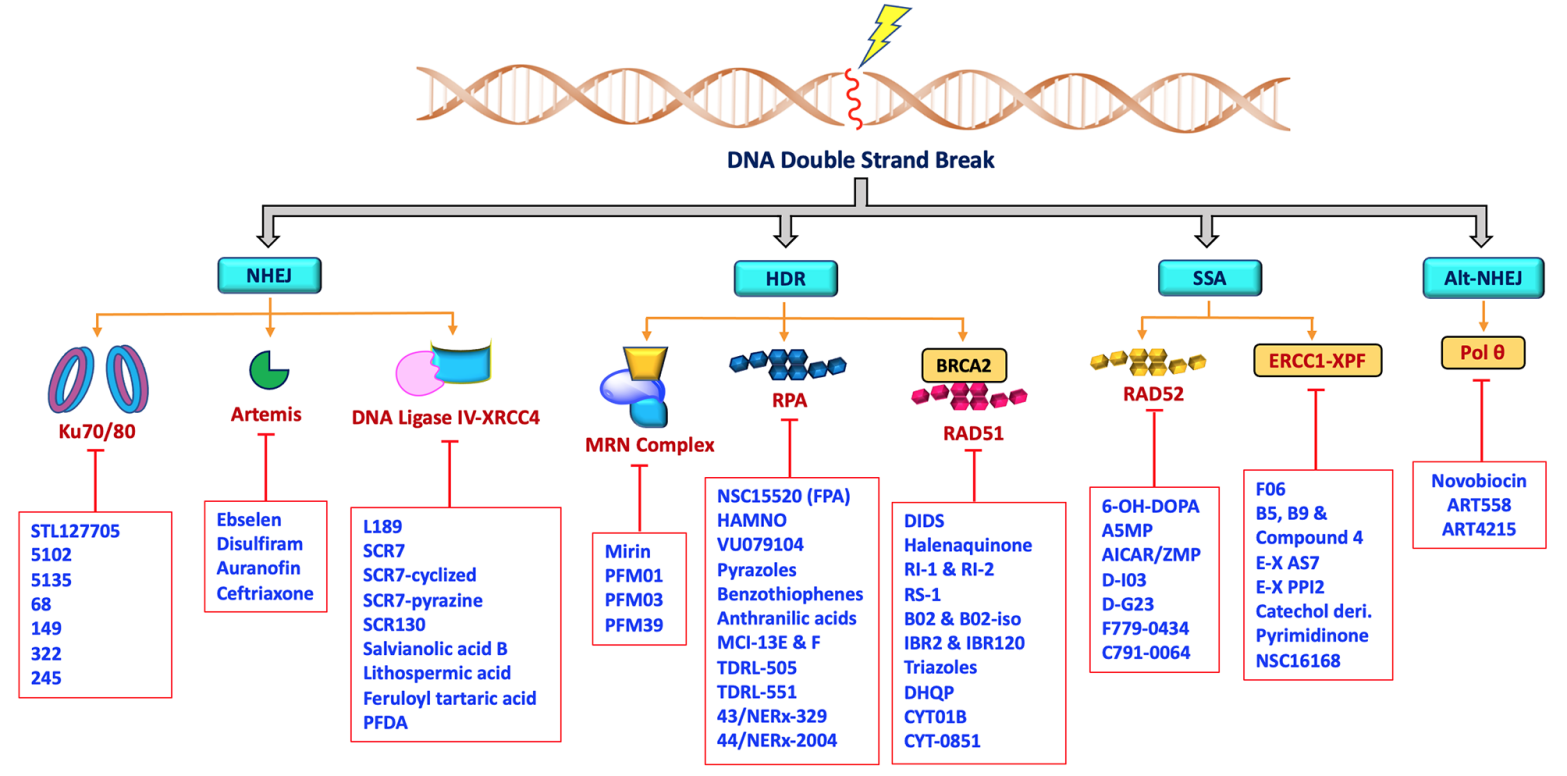
Schematic representation showing non-PIKKs DSB repair inhibitors that target key/core and accessory proteins involved in DSB repair pathways.

The rare mutations and altered expression levels of key NHEJ and HDR proteins, mainly Ku70/80, DNA-PK, Artemis, Ligase IV, XRCC4, XLF, MRE11, RAD51, RPA and RAD52 can lead to predisposition to cancer, whereas increased capacity of DNA repair and DDR can be clinically exploited by targeting repair pathways to overcome resistance and enhance chemo- or radiosensitivity in cancer patients (5, 71–75). DNA repair inhibitors can be used to specifically target proteins involved in key steps of NHEJ, HDR, MMEJ and SSA as well as core or processing proteins involved in DDR signaling pathways. Developing drugs aimed at modulating DNA DSB repair activity are most likely to have a profound impact on the efficacy of radio- and chemotherapy. Therefore, targeting these key proteins in the DNA DSB repair pathways (**Figure 2**) has recently become a popular approach for potential cancer treatments.

## Inhibitors Targeting NHEJ Pathway

### Ku 70/80 Inhibitors

There has been considerable progress made in the development of DNA-PK inhibitors and several of them are in various stages of clinical trials (NCT02644278, NCT04172532, NCT03907969), but less attention has been placed on the upstream and most essential Ku70/80 heterodimer that recruits DNA-PKcs (68, 69). In the absence of heterodimeric Ku subunits, DNA-PKcs binding affinity to DNA DSB is significantly weak, resulting in halting of the repair process (76). DNA-PK has a unique mechanism of activation that requires binding to DNA termini, and this strong binding interaction is solely dependent on a protein-protein interaction with the Ku70/80 heterodimeric complex for the subsequent NHEJ activation (77, 78). Being the primary sensor and core regulator of this pathway, Ku is absolutely required for DNA DSBs repair by NHEJ (79–81). Inhibition of Ku subunits could therefore produce reduced DNA-PK and NHEJ activity. Therefore, Ku has a high potential for therapeutic outcomes in oncology.

**Figure 3:**
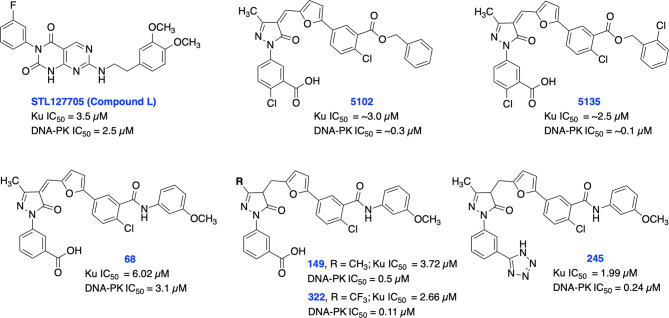
Small molecule inhibitors of Ku70/80 and their respective IC50 values for disruption of DNA-binding by Ku70/80 and DNA-PK activity.

Recent studies have demonstrated a significant increase in expression levels of Ku70 and Ku80 after chemo- and radiotherapy which correlates with poor prognosis in patients with rectal and cervical cancers (82–84). Further studies have also demonstrated that overexpression of Ku70/Ku80 is directly correlated with chemotherapy and radiotherapy resistance in various cancers (82). Previously, shRNA depletion of Ku70 or Ku80 produced cytotoxicity and radiosensitization in pancreatic cancer cells (85). In addition, Ku70 or Ku80 null cells exhibited enhanced chemo sensitization to DNA damaging agents including bleomycin, doxorubicin, and etoposide (86). Ku is also involved in several other DNA metabolism processes and in telomere maintenance (35, 36, 87). Despite the crucial role of Ku subunits early in the NHEJ pathway, there are currently a limited number of Ku70/80 inhibitors developed so far. In 2016, Weterings et al. identified STL127705 (compound L) (**Figure 3****)** by computational screening of a commercial library that disrupts Ku-DNA binding activity in micro-molar range and has potential to sensitize cancer cells to IR (88). However, the ability of STL127705 to block NHEJ catalyzed DNA DSB repair is not documented to date.

**Figure 4:**
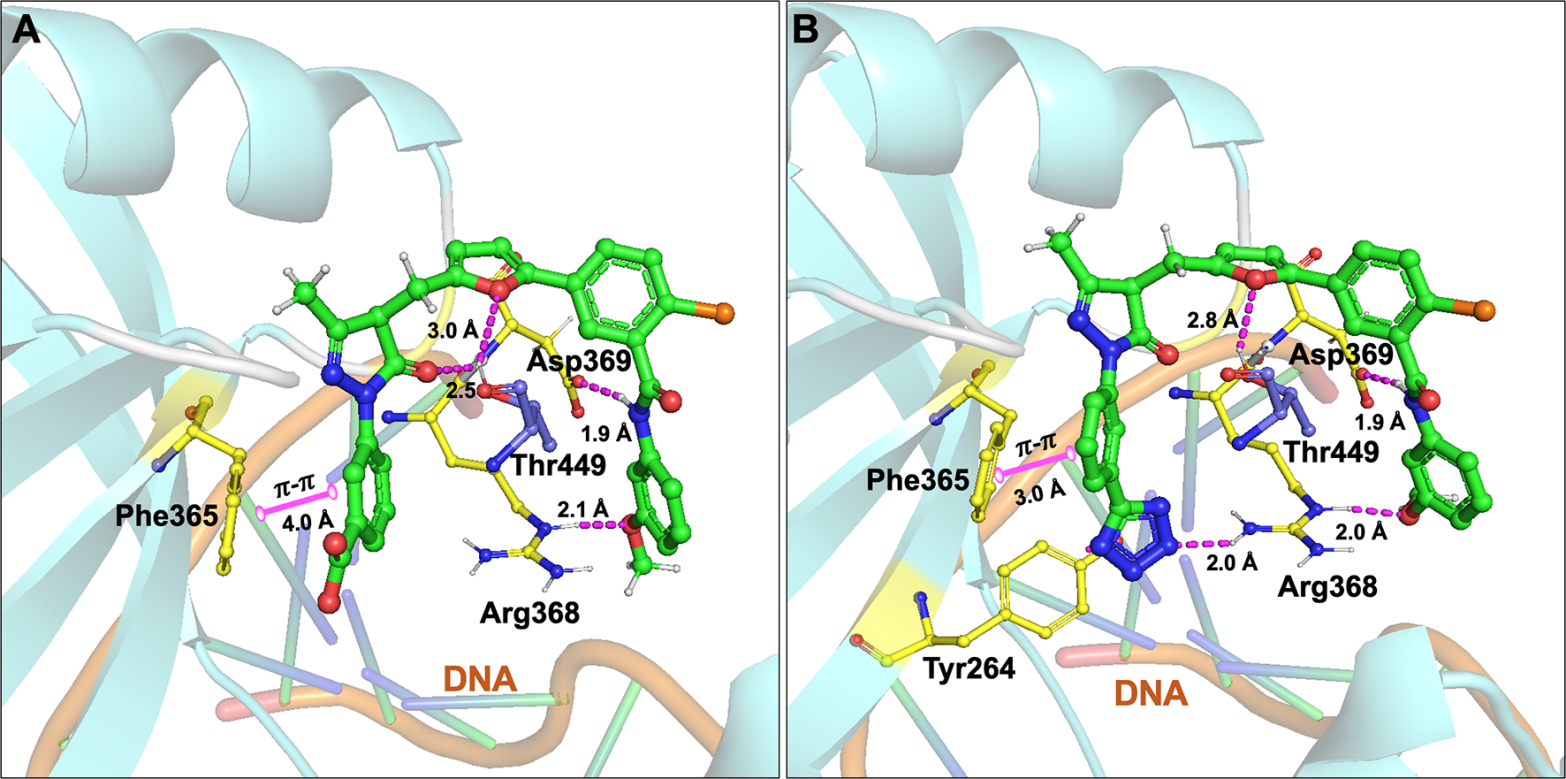
Molecular interactions of **(A)** compound 149 and **(B)** 245 (all in green carbon) with Ku70/80 heterodimer (key amino acids are shown in yellow carbon (Ku70), blue carbon (Ku80) and cartoon is shown in cyan color). Interaction with amino acid side chains is indicated with the dashed magenta lines and π – π stacking interactions are shown in solid magenta dumbbell. The DNA helical structure is depicted in greenish blue sticks and light orange cartoon. Interaction distances indicated in Å.

Initially, our group identified arylalkyl esters of arylpyrazolone carboxylic acid derivatives, 5102 and 5135, through screening of a commercial library and both inhibitors displayed high potency in both Ku-DNA EMSA and DNA-PK kinase assays (**Figure 3**) (89). Retaining the core scaffold employed in 5102 and 5135, we recently further expanded our structure-guided synthetic chemistry efforts with the aim of improving Ku inhibitory potency, selectivity, and cellular activity while simultaneously improving solubility among other physicochemical properties (90). The structure activity relationship (SAR) from this study showed that an amide moiety increased both the solubility and the inhibition of Ku-DNA interaction by 4-fold over the ester group. Compounds 68, 149, 322 and 245 exhibited a high potency and specificity towards Ku and DNA-PK. Moreover, these compounds also showed improved chemical properties including solubility and stability. These Ku-DNA binding inhibitors (Ku-DBi’s) directly interact with Ku and inhibit *in vitro* NHEJ, cellular NHEJ, and potentiate the cellular activity of radiomimetic agents and IR. Further analysis demonstrated that Ku-null cells are insensitive to Ku-DBi’s however, Ku-DBi’s potentiate cellular sensitivity to DSB-inducing agents in cancer cells. Molecular docking studies indicated that compounds 149 and 245 possess high affinity towards the Ku binding site (**Figure 4**). Inhibiting Ku interactions with DNA ends can efficiently block NHEJ catalyzed repair which is anticipated to increase efficiency of HDR-mediated recombination events. Therefore, we performed CRISPR-Cas9-mediated genome editing in the presence of Ku-DBi 245 where we observed a 6-fold increase in HDR mediated insertion at a DSB at the target site compared to the controls (90). These data suggests that Ku-DBi’s could be effective to reduce off-target, potentially mutagenic events that have hampered CRISPR mediated therapeutic applications.

Further development of Ku70/80 inhibitors has a considerable potential to impact cancer therapy as well as precise genome editing.

### Artemis Inhibitors

Artemis is a structure specific endonuclease with critical roles in DSB repair by NHEJ, in the development of B- and T- lymphocytes *via* cleaving a hairpin intermediate during V(D)J recombination and has also been implicated to play a role in the maintenance of genomic stability (91–94). Artemis was first reported after investigators implicated its deficiency in severe combined immunodeficiency (SCID) as causative for observed phenotypes in this disorder including impaired V(D)J recombination and enhanced IR sensitivity, supporting the mechanistic role of Artemis in these pathways. Mouse embryonic fibroblasts (MEFs) derived from Artemis defective mice have increased chromosomal abnormalities, suggesting a role for Artemis in genome stability maintenance (95). In NHEJ, DNA-PKcs undergo autophosphorylation and activate the endonuclease activity of Artemis at DNA ends (93). Artemis’ C-terminal region influences V(D)J recombination through its interactions with DNA Ligase IV and DNA-PKcs, suggesting that the Artemis-binding site on Ligase IV also has physiological relevance to potentially disrupting NHEJ complex formation (96, 97). Artemis is the main nuclease known to remove DNA single-strand overhangs and 3′-phosphoglycolate groups from DNA termini generated by IR with its endonuclease activity (98). It is well documented that IR-induced DSBs require Artemis for repair (94, 95, 99, 100).

**Figure 5:**
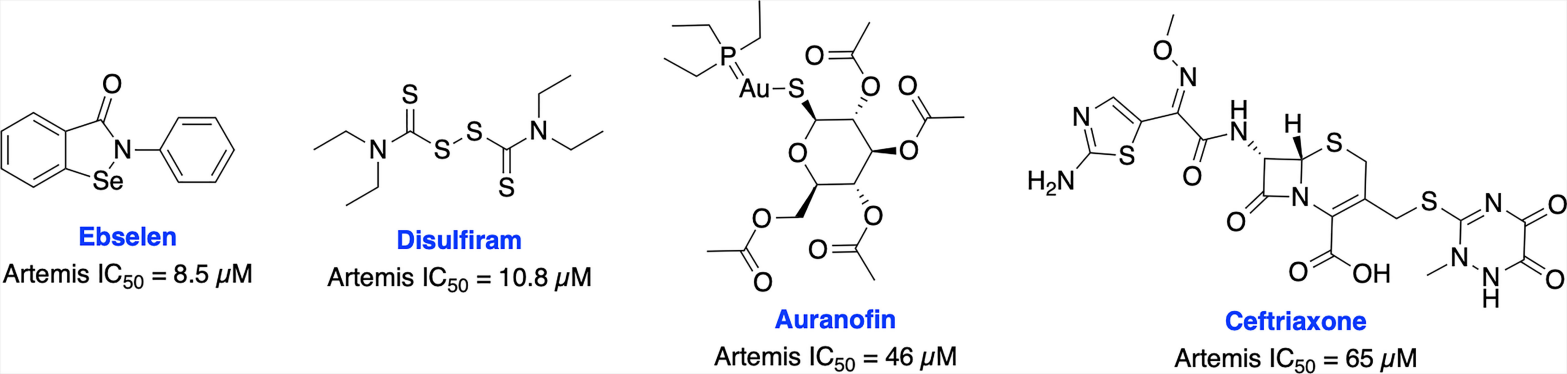
Small molecule inhibitors targeting Artemis and their respective IC50 values for disruption of endonuclease activity.

Recently, Yosaatmadja et al. generated a model for Artemis DNA binding based on their zinc bound Artemis crystal structure and another recently reported structure of the Artemis catalytic domain (101, 102). This unique zinc-finger-like motif has not been reported in other metallo-β-lactamase (MBL) enzymes (the super family to which Artemis belongs) and presents a possible novel targeting location. Further, they have screened thiol reactive compounds using this unique zinc-finger like motif of Artemis and identified that ebselen and disulfiram are able to inhibit Artemis endonuclease activity in the low micro-molar range (IC_50s_ = 8.5 uM and 10.8 uM, respectively), while auranofin and ceftriaxone are less potent (IC_50s_ = 46 uM and 65 uM, respectively) (**Figure 5**).

The recent crystal structures of Artemis and these inhibitors provide useful information for structure-based design of inhibitors to generate more selective and potent Artemis inhibitors, either binding at the active site or the unique zinc finger motif of Artemis. The key roles of Artemis within DNA repair make it an attractive target for a variety of therapeutic avenues. Artemis inhibitors have the potential to be used as radiosensitizers in various tumor types, demonstrated biologically by the sensitivity to IR seen in SCID patients. Because of its clear role in DNA repair and genome stability, Artemis targeted inhibitors also have the potential to synergize well with other DDR targeted inhibitors. There is the potential for impacts on immune cell maturation with long term clinical Artemis inhibition which could result in compromised immune function, thus monitoring immune system function will be critical as Artemis targeted agents progress to the clinic.

### DNA Ligase IV Inhibitors

After DNA end processing, the final step in NHEJ pathway is ligation which is a crucial step in the repair of DNA DSBs and is an attractive target for inhibition of the DSB repair pathway. This is demonstrated by various Ligase IV deficient mutants and knockout studies, that have been shown to have significantly reduced NHEJ activity (103–105). Upon activation of kinase activity by DNA-PKcs, Ku heterodimer translocates internally to make DSB ends accessible to a specific ligation complex, which is composed of DNA ligase IV and its partnering proteins, XRCC4 and XLF (106).

**Figure 6:**
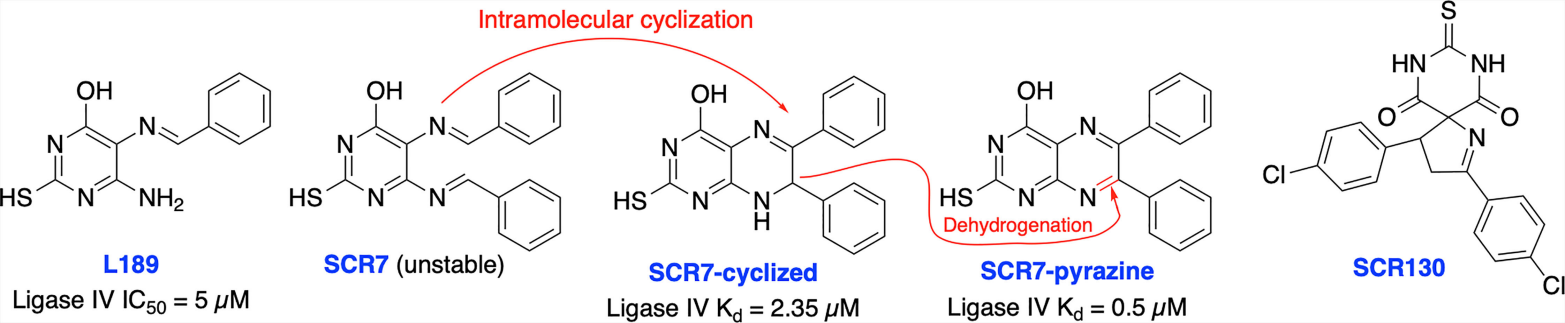
Small molecule inhibitors targeting DNA Ligase IV and their IC50 values for either inhibition of Ligase IV adenylation or Ligase IV end-joining.

In 2008, Chen et al. identified a competitive and non-specific ligase inhibitor, L189 through a computational drug design strategy which showed equipotent inhibitory activity against Ligase I, III, and IV (**Figure 6****)** (107). Raghavan and co-workers in 2012 developed SCR7, a derivative of L189, which was initially suggested to be more selective for Ligase IV (108). Further extensive structural analysis by Greco et al. revealed that parental SCR7 is nonspecific, only exists in the more stable cyclized SCR7 pyrazine form and failed to inhibit DNA ligase IV-dependent V(D)J recombination in a cell-based ligation assay (109). On the contrary, Raghavan and co-workers in 2018 showed both intramolecular cyclized SCR7 (SCR7-cyclized) and further oxidized product (SCR7-pyrazine) could inhibit Ligase IV-mediated end joining and V(D)J recombination (110). Further studies showed that the SCR7-cyclized is Ligase IV specific and SCR7-pyrazine induced nonspecific cytotoxicity at higher concentrations in Ligase IV-null cells. Recently, Raghavan and co-workers developed a new ligase IV-specific inhibitor, SCR130, which exhibited 20-fold improved cytotoxicity compared to SCR7 and potentiated radiosensitivity in cancer cells (111). Furthermore, SCR7 produced enhanced HDR-mediated repair for CRISPR mediated genome editing by inhibiting NHEJ at lower concentrations (1 μM) (103), but cellular toxicity was observed with concentrations above 1 μM (104), suggesting that cell-dependent toxicity or off-target effects associated with the inhibitor (112). The higher IC_50s_ and inconsistent results could be explained by instability of parental SCR7 and its analogs. Given the conflicting results and unclear therapeutic and toxicological mechanisms of action, more research is required on this area. There is also a great need within medicinal chemistry to identify novel scaffolds apart from SCR7 to target Ligase IV as this will broaden the chemical space available to develop Ligase IV inhibitors.

### XRCC4 Inhibitors

**Figure 7:**
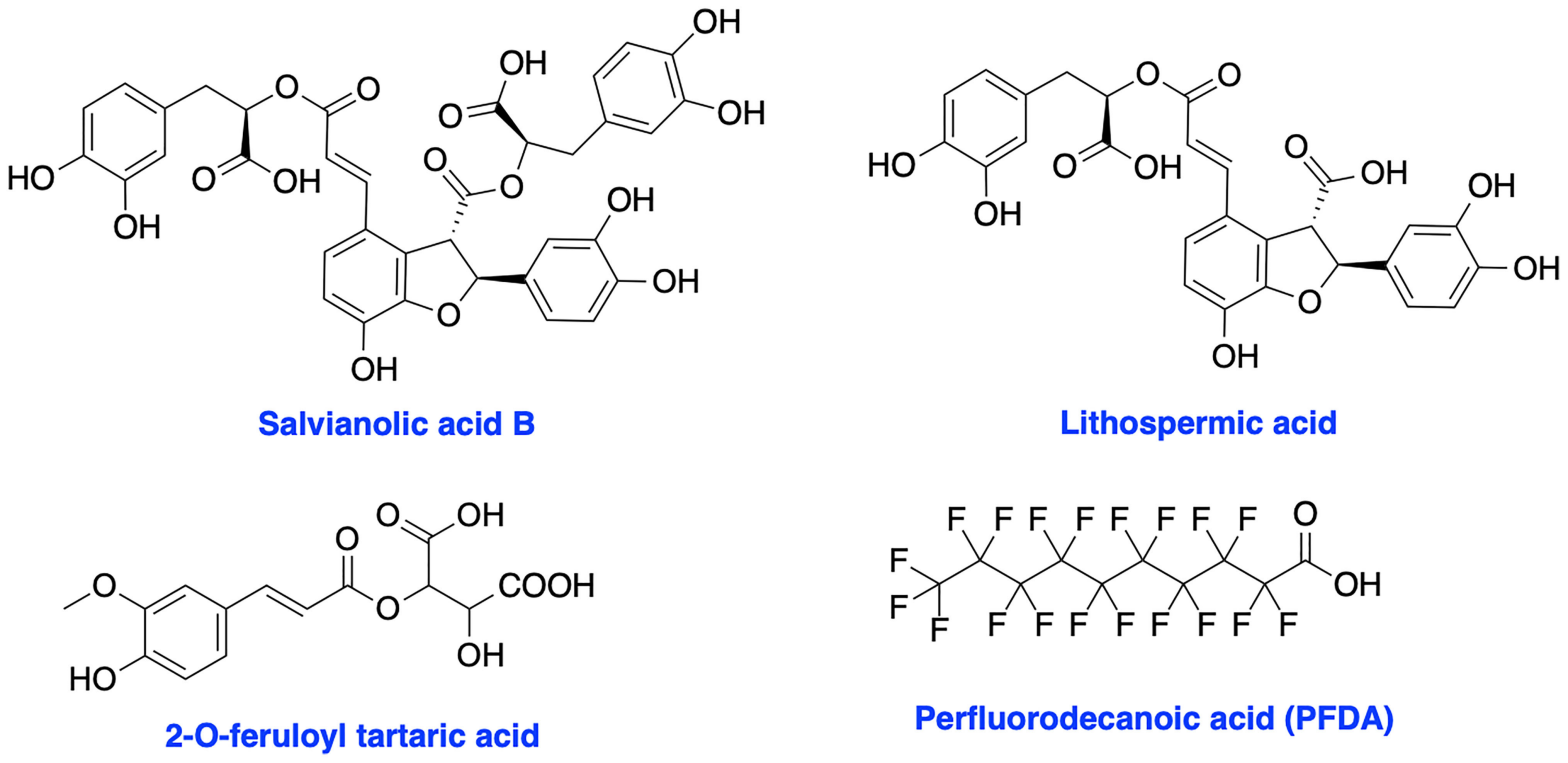
Structures of XRCC4 inhibitors.

XRCC4 and its paralog, PAXX are responsible for the recruitment of other NHEJ factors to the damage site and XRCC4 is also a key regulator of DNA ligase IV activity in the NHEJ ligation step (17, 113–115). XRCC4 holds a potential to enhance chemo- and radiosensitivity of current therapeutics. Early attempts to inhibit XRCC4 resulted in the development of compounds salvianolic acid B, lithospermic acid, and 2-*O*-feruloyl tartaric acid (**Figure 7**); however, potential *in vitro* and *in vivo* effects of these agents is not documented to date (116). Recently, Liu et al. identified perfluorodecanoic acid (PFDA), a common persistent environmental pollutant, as a XRCC4 inhibitor which was able to sensitize gastric cancer cells to chemotherapy; however, mechanism of action, target engagement with XRCC4 and the toxicity profile of the inhibitor needed to be explored in more details (117).

## Inhibitors Targeting HDR Pathway

### MRN Complex (MRE11-RAD50-NBS1) Inhibitors

In case canonical NHEJ pathway fails to enact timely DNA repair, DSBs are subjected to end resection leading to the generation of 3′ ss-DNA that interfere with Ku recruitment and promote high-fidelity repair process by HDR (11, 118). Homologous recombination occurs between homologous DNA sequences through the MRN-RPA-RAD51 axis which facilitates repair of the damaged sequence without loss of genetic information. The DSB recognition and DNA end resection are mediated by MRE11-RAD50-NBS1 (MRN) complex which further recruits and activates ATM kinase immediately after detection of DSB. Simultaneously, RPA mediates the recruitment of ATR/ATRIP (11, 119–121). An additional oncogenic role of the MRN complex involves promoting telomere lengthening *via* alternative telomere lengthening (ALT) by homologous recombination (122, 123). In this case, the chromosomal ends are first resected 5’→3’ and then treated as broken ends for HDR. Additional mechanistic insight into HDR in response to telomeric DSBs and telomere lengthening has recently been reported (124).

The crucial role of the MRN complex in DSB repair and its potential as a target for cancer therapy has been widely explored in various types of cancers. The high-level expression of MRN complex is associated with chemo- and radio-resistance in breast cancer, glioblastoma and NSCLC as well as correlated with worse disease-free (DFS) and poor overall survival (OS) in rectal, prostate, gastric and NSCLC patients. However, the consequences of defects and/or altered expression level of MRN complex are still controversial with respect to its dual roles in tumorigenesis and prognosis (125–128).

**Figure 8:**
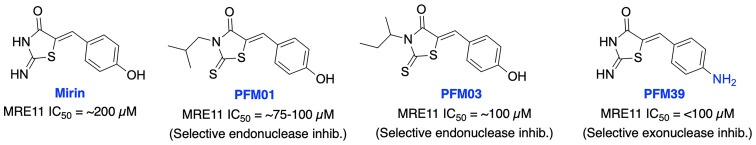
Small molecule inhibitors targeting MRE11 with their respective IC50 values for inhibition of nuclease activity.

Initial attempts to inhibit MRE11 resulted in Mirin as the first MRE11 inhibitor from high-throughput screening (HTS). Mirin blocks Mre11 exonuclease activity, prevents MRN-dependent ATM activation without affecting its kinase activity and abolishes the G2/M checkpoint and homology-dependent repair in mammalian cells (129). Mirin displayed inhibition of androgen-dependent transcription and growth of prostate cancer cells, MYCN-amplified neuroblastoma cells and enhanced chemosensitivity to DNA damaging agents in glioblastoma cells (130–132). Further structural modification of Mirin resulted in PFM01 and PFM03 as selective endonuclease inhibitors and PFM39 which selectively block the exonuclease activity of MRE11 (**Figure 8**), while their potential function in cancer therapy remains poorly explored (133). Further mechanistic studies revealed MRE11 exo- or endonuclease inhibitors confer distinct DSB repair mechanisms. Inhibition of endonuclease activity of MRE11 drives the cell to NHEJ repair pathway over HDR, while blocking the exonuclease activity of MRE11 results in a repair defect. These studies demonstrate the potential impact of targeting MRN complex for cancer therapy; however, the lack of HDR specificity and the broad spectrum of activity restricted further development of these inhibitors.

To date, there is no inhibitor developed targeting RAD50 and NBS1 despite their crucial role in MRN complex mediated repair pathway and targeting protein-protein interactions in the MRN complex could also provide a potential chemotherapeutic strategy.

### RPA Inhibitors

Replication protein A (RPA) is the major human single stranded DNA (ssDNA)-binding protein and plays critical roles in a variety of DNA metabolic pathways including DNA replication, repair, recombination, checkpoint activation and DDR. RPA interacts with several functional proteins to regulate DNA metabolism for the maintenance of genomic stability. RPA’s integral and non-redundant roles in both nucleotide excision repair (NER) and homology directed repair (HDR) DNA repair pathways have been well studied. Beyond NER and HDR, RPA is involved in the process of replication fork reversal and other DNA maintenance pathways such as DNA mismatch repair (MMR) and base excision repair (BER) (134–137). In NER, the recognition and verification of bulky adduct DNA damage requires RPA in conjunction with XPA while in HDR, RPA ssDNA-binding activity is required to promote RAD51 filament formation in preparation for strand invasion. RPA binding drives a chain of cooperative events that results in the recruitment of HDR repair proteins (including BRCA1 and BRCA2) at the site of DSB DNA damage. RPA acts as a key sensor to elicit cell cycle arrests at checkpoints and potentiate the activation of the ATR kinase mediated DNA damage signaling/DDR by following cellular exposure to genotoxic stresses (135, 138). Each of these roles requires binding of RPA to ssDNA, making the RPA-ssDNA interaction a promising target for cancer therapy. RPA has been shown to be over-expressed in several cancers including lung, ovarian, breast, colon, bladder, gastric, hepatic, and esophageal and these solid tumors may rely on RPA to mitigate the replication stress associated with these cancers (135, 139, 140).

**Figure 9:**
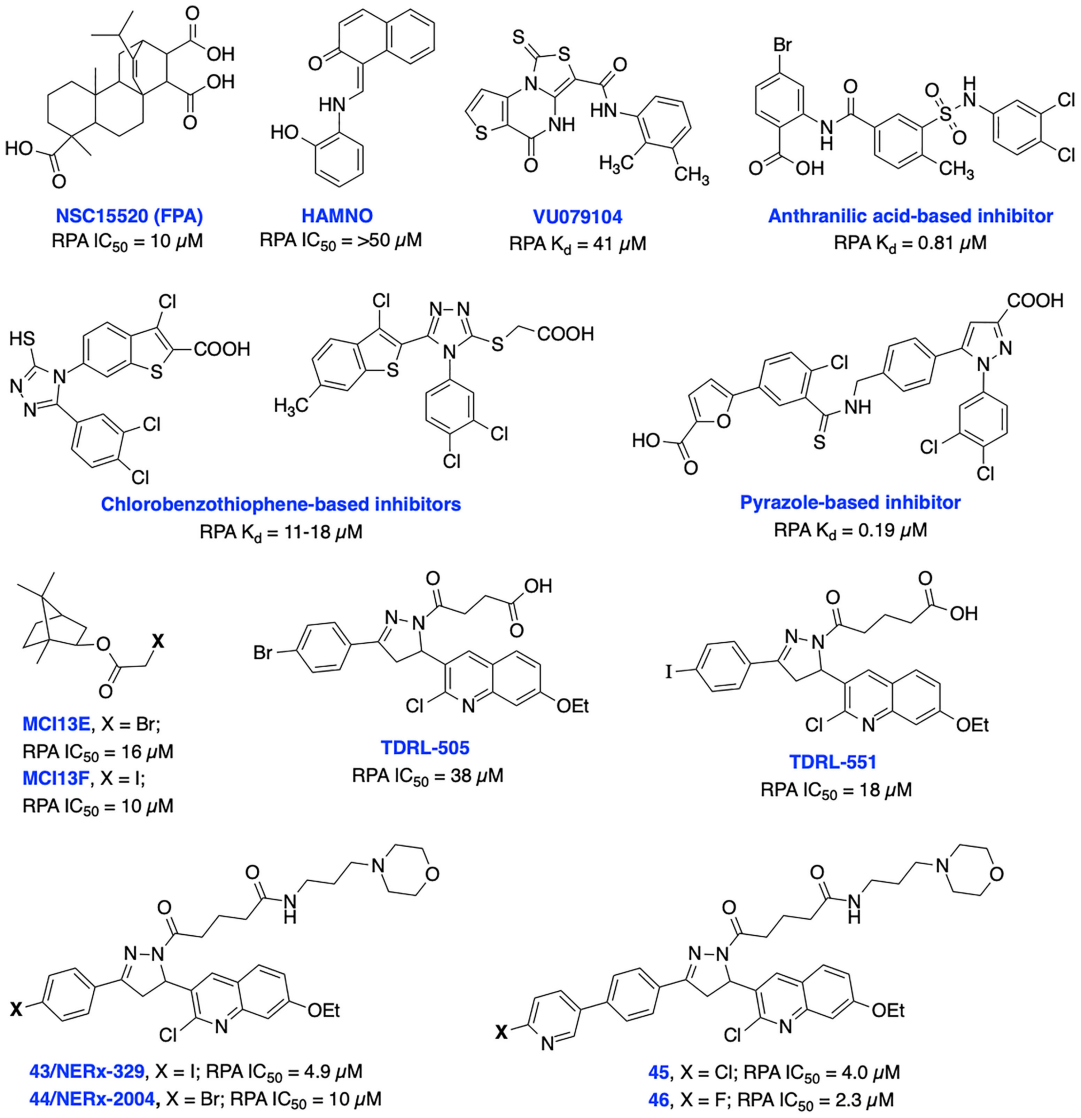
Small molecule inhibitors targeting RPA N-terminal protein-protein interactions and RPA-DNA interactions with their respective Kd/IC50 values.

RPA is a heterotrimeric complex consisting of 70 kDa (RPA70), 32 kDa (RPA32), and 14 kDa (RPA14) subunits (141). The 70 kDa subunit contains the two major high affinity ssDNA binding domains A and B, in addition to domains C and F. The F-domain located on the N-terminal of the 70 kDa subunit (RPA70N) of RPA does not bind ssDNA with high affinity; however, it is involved in a series of protein–protein interactions. The development of small molecule inhibitors of RPA has been pursued by either targeting the (i) N-terminal region of the 70 kDa subunit (RPA70N) to disrupt its interactions with key DDR proteins or (ii) the DNA-binding A and B domains of RPA to prevent binding of ssDNA. Early attempts to develop N-terminal RPA70N targeted inhibitors resulted in NSC15520 (Fumaropimaric acid, FPA) and HAMNO (**Figure 9**); however, their further progress is restricted due to limited cellular uptake, specificity, or metabolic instability (142–144). Fesik and co-workers exploited fragment-based NMR, HTS screening approaches, and further structure-based optimization efforts which led to the discovery of nanomolar or sub micromolar stapled helix peptides, thiazolothienopyrimidinone- (VU079104), anthranilic acid-, chlorobenzothiophene-, pyrazole-based inhibitors targeting RPA70-N-terminal domain (62, 137, 145). High binding affinity, good *in vitro* potency and cellular uptake observed with some of these inhibitors suggest potential for further development, albeit neither cellular activity nor specificity is documented to date.

Recent advances in the development of inhibitors targeting protein-DNA interactions hold considerable promise and opened an entirely new class of ‘druggable’ targets for therapeutic intervention. Earlier, we identified isoborneol haloacetate MCI13E and MCI13F as potent RPA inhibitors and biochemical analysis revealed an irreversible mechanism of inhibition involving covalent modification of RPA with these inhibitors. MCI13E showed cytotoxicity, induced apoptosis and demonstrated synergy with cisplatin in lung cancer cell line models (62, 146). Toward identifying reversible inhibitors of the RPA-DNA interactions, we identified TDRL-505 through HTS screening using a fluorescence polarization-based assay (147, 148). Further SAR studies with TDRL-505 scaffold generated several analogs and among them TDRL-551 was identified as the most potent compound (149). This proof-of-concept study identified that both inhibitors were capable of blocking the RPA-DNA interaction, resulting in cell cycle arrest, cytotoxicity, and increased the efficacy of the chemotherapeutic drugs cisplatin and etoposide *in vitro*. Moreover, TDRL-551 displays modest single agent activity in lung and ovarian cancer cell lines and synergy in combination with cisplatin and etoposide. Recently, we performed systematic structural modification of TDRL-551 in our laboratory by utilizing a structure-based drug design strategy and identified a series of novel chemical inhibitors (43/NERx-329, 44/NERx-2004 and 45-46) with improved RPA inhibitory potency, solubility, and cellular uptake for preclinical settings (150). Moreover, NERx-329 exhibited single agent activity in a broad spectrum of cancer cells, synergism with DNA damaging agents (cisplatin, etoposide and bleomycin) and DDR inhibitors (BMN673, NU7441 and VE821) in lung cancer cells and single agent anticancer activity in lung cancer xenograft models. DNA fiber analysis showed degradation of replication forks upon stalling and RPA exhaustion by NERx-329 and other known DDR inhibitors (151). Overall, a multifaceted role of RPA mediated DNA damage repair through NER, DSB repair through HDR, DNA damage signaling/DDR, replication fork dynamics and its interaction with other proteins holds the potential to fine tune the pathway and it’s response to chemotherapy or radiotherapy induced DNA damage toward maximizing efficacy, overcoming resistance, and reducing the toxicities associated with existing cancer therapeutics.

### RAD51 Inhibitors

RAD51 is essential for promoting the HDR pathway as RAD51 binds to ssDNA by displacing RPA with the help of BRCA2 and other accessory factors to allow homology search and strand invasion.

Both RAD51 and RPA also are essential regulators of replication forks stability including in regulating fork restart and reversal through management of ssDNA. RAD51 and RPA function early in the processing of stalled forks, before the formation of a DSB, to facilitate fork reversal and protection that help maintain genome stability during DNA replication (152, 153, 136). RAD51 overexpression is observed in several cancers, including pancreatic, soft tissue sarcoma, breast, NSCLC, prostate cancer, glioblastoma and leukemia (152). Overexpression of RAD51 enhanced DNA repair HDR activity and helps cancer cells to survive and develop resistance to DNA damaging agents (154, 155). Depletion of RAD51 expression or inhibition heightened sensitivity to DSB inducing agents including IR in various cancer cells. Therefore, developing RAD51 inhibitors could lead to persistent DNA damage, G2/M arrest, apoptosis in the cancer cells and overcome resistance associated with current DSB inducing agents. Additionally, making HDR-proficient tumor cells HR-deficient by inhibiting RAD51 could prove useful in restoring synthetic lethality in tumors that have developed resistance with PARP inhibitors (PARPi).

**Figure 10:**
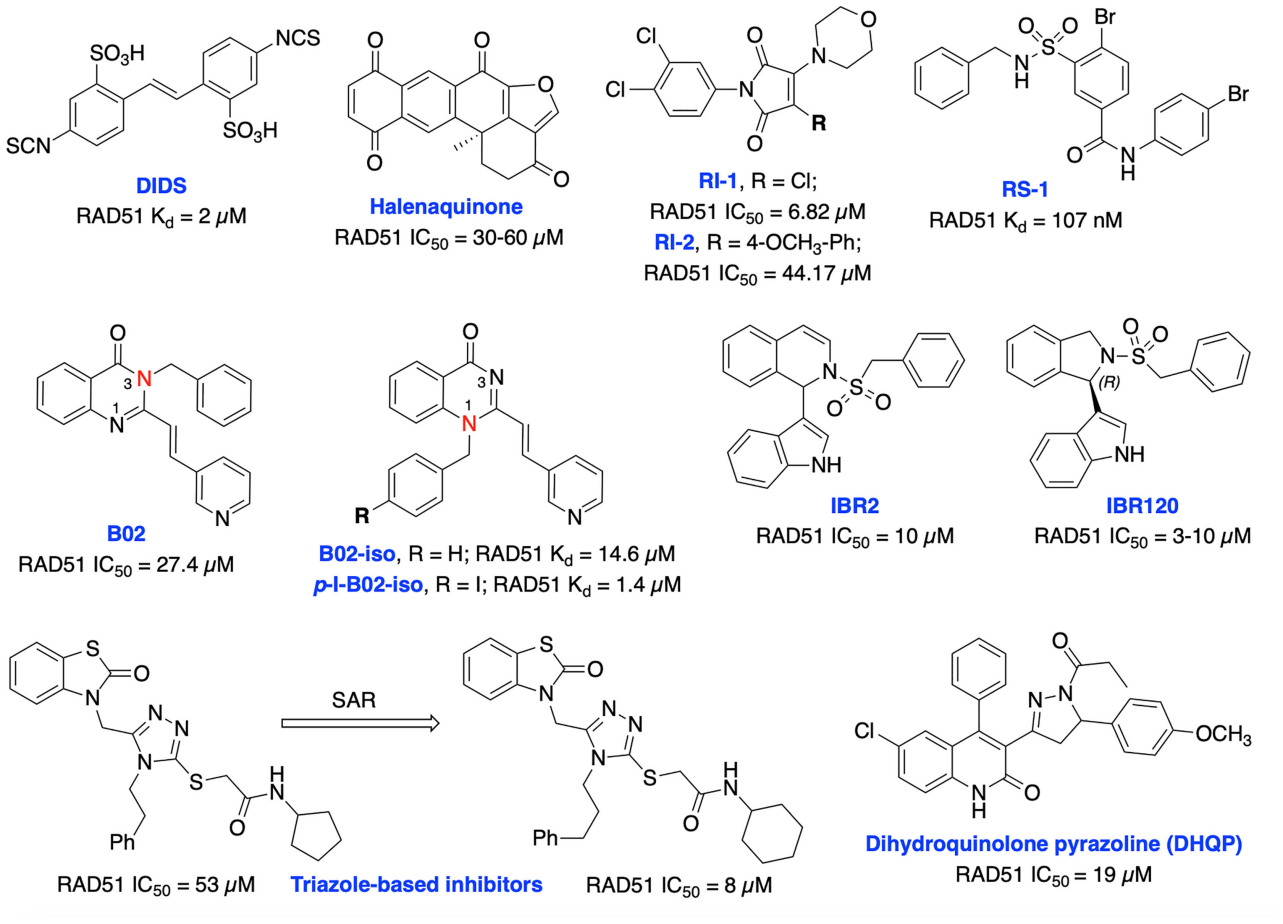
Small molecule inhibitors targeting RAD51 with their respective Kd/IC50 values for either disruption of RAD51 binding or RAD51 mediated D-loop formation.

Currently, several RAD51 inhibitors have been developed to further exploit the HDR pathway as a therapeutic target for cancer therapy. RAD51 has been explored as a pharmacological target in two different ways, first, in cancers known to overexpress RAD51, compounds with single-agent activity have been described that exploit overexpression by inducing formation of toxic RAD51 complexes on undamaged DNA. The second of which is as a component of combination therapy where disruption of RAD51’s ssDNA binding activity synergizes DNA damaging therapies. Ishida et al. identified DIDS as a competitive RAD51 inhibitor that prevents RAD51-ssDNA and RAD51-dsDNA binding, RAD51-mediated strand exchange and homologous pairing (**Figure 10**). However, the elevated human cell toxicity of DIDS has restricted its further development (156). A natural compound, halenaquinone was identified through an extensive screen of marine sponge extracts which directly inhibit RAD51-dsDNA binding, but it does not alter RAD51 affinity for ssDNA (157). Furthermore, halenaquinone-treated cells showed a reduction of IR induced RAD51 foci formation at DSB sites probably by preventing the DNA homologous pairing step of the HDR pathway. Chloromaleimide derivative RI-1 was identified as a potent RAD51 inhibitor and its biochemical analysis revealed an irreversible mechanism of inhibition involving covalent modification of the thiol group on the C319 residue of human RAD51 (158). In order to avoid off-target effects associated with covalent inhibitors and improve metabolic stability of the compound in biological systems, the reversible RAD51 inhibitor RI-2 was developed by introducing an aromatic ring at maleimide ring. RI-2 displayed a 6-fold decrease in potency compared to RI-1 and specifically inhibited HDR efficiency and sensitize human cancer cells to mitomycin C (MMC)-induced synthetic lethality (159).

The small molecule RS-1 was developed as an allosteric effector to exploit overexpression of RAD51 activity by further stimulating the formation of toxic RAD51 complexes on undamaged chromatin as a potential cancer therapy (160, 161). RS-1 was able to stimulate binding of RAD51 to ssDNA and dsDNA and enhanced recombination activities of RAD51 by locking its active conformation, without affecting ATP hydrolysis. RS-1 demonstrated a single agent activity in tumor cell lines which have more ssDNA due to increased replication, that leads to cytotoxicity while sparing normal cells (162). RS-1 also showed significant anticancer activity in a prostate cancer xenograft animal model (161). RS-1 exhibited inconsistent HDR efficiency in CRISPR/Cas9 precision genome editing in various other organisms and cell types (163, 164), suggesting that either different species may respond differently, or RAD51 may not be the most reliable target for improving precision genome engineering applications. Mazin and co-workers identified B02 as a highly specific RAD51 inhibitor that directly binds to RAD51, increases sensitivity to IR and several DNA damaging agents including etoposide and doxorubicin by inducing DSBs and subsequent blocking of HDR repair (154, 165, 166). Recently, they have carried out further structural analysis of B02 and identified B02-iso and *p*-I-B02-iso as substantially stronger inhibitors of RAD51 and HDR than the parent compound. B02-iso significantly increased the sensitivity of BRCA-proficient triple-negative breast cancer (TNBC) MDA-MB-231 cells to the PARPi, olaparib through synthetic lethality (167).

Zhu et al. targeted protein–protein interaction sites of RAD51 by developing IBR2 which disrupts the RAD51-BRCA interaction and RAD51 multimerization and enhances proteasomal degradation of RAD51 (168). Further structural optimization of IBR2 generated the stereo selective inhibitor IBR20 which also disrupts RAD51 multimerization, impairs HDR activity and increases cytotoxic activity in a variety of cancer cell lines (169). Utilizing high throughput docking and further SAR optimization, Cavalli and co-workers identified a series of triazoles that mimic BRCA2 mutations by disrupting the RAD51-BRCA2 interaction. Further, these compounds inhibited DSB repair and exhibited synergy with olaparib in pancreatic cancer cells with functional BRCA2 (170, 171). Recently, the same research group identified a dihydroquinolone pyrazoline (DHQP)-based inhibitor which also disrupted the RAD51-BRCA2 interaction, inhibited HDR activity and showed synergy with olaparib in pancreatic cancer to trigger synthetic lethality (172). However, further structural optimization is needed to improve potency, solubility, cytotoxicity and true synthetic lethality outcome of both triazole- and DHQP-based inhibitors. The fatty acid nitroalkene 10-nitro-octadec-9-enoic acid (OA-NO_2_) inhibited RAD51-ABL1 complex formation by alkylating RAD51 Cys-319 residue and decreased HDR activity. It also increased the sensitivity of doxorubicin, olaparib, IR and cisplatin in TNBC cells (68, 173). CYT01B and CYT-0851 (structures are not disclosed), are orally bioavailable small molecule RAD51 inhibitors, being developed by Cyteir Therapeutics. Both inhibitors blocked HDR activity and have demonstrated anticancer activity in cells expressing activation-induced cytidine deaminase (AID), a protein that promotes formation of DSBs. Preclinical data showed synergy with PARP and ATR inhibitors in various models, suggesting these inhibitors have the ability to overcome resistance of PARPi (174, 175). CYT-0851 is currently in Phase 1/2 clinical trials demonstrated promising antitumor activity *in vitro* and *in vivo* models across different tumor types including both hematologic malignancies and solid tumors (NCT03997968, https://clinicaltrials.gov/ct2/show/NCT03997968).

The development of either RAD51 inhibitors or modulators can be safe and effective for clinical use and it is an exciting approach for cancer therapy.

## Inhibitors Targeting SSA and Alt-NHEJ (TMEJ) Pathways

SSA is a RAD51-independent DSB repair pathway which joins two homologous repetitive sequences oriented in the same direction through annealing. SSA shares DNA end resection and RPA displacement steps with HDR to reveal complementary homologous sequences. RAD52 is the central protein for SSA which is recruited to anneal each ssDNA with two repetitive sequences. After the annealing step, the sequences between the homologous repeats are flanked out on either side. These flanked ends are then cleaved off by nucleases, preferentially by ERCC1/XPF endonuclease and finally the ssDNA gap is closed by ligation (11, 176).

Alt-NHEJ (MMEJ/TMEJ) utilizes short microhomologies to join the two DNA strands. PARP1 is involved in promoting DNA end synapsis and recruiting the DNA polymerase θ (Pol θ) to DSB ends. Pol θ eventually stabilizes microhomology-mediated joints between the two DNA ends and flaps extending from these joints are cleaved off by either ERCC1-XPF or Flap endonuclease 1 (FEN1), followed by a ligation step (5). However, both SSA and alt-NHEJ DSB repair pathways serve primarily as backup pathways in mammalian cells which are deficient of either NHEJ or HDR pathways.

### RAD52 Inhibitors

RAD52 plays essential roles in homology dependent DSB repair. RAD52 binds to ssDNA, promotes DNA annealing in the SSA pathway while it interacts with RAD51 to modulate its DNA strand-exchange activity in the HDR pathway. In addition, RAD52 protects stalled replication forks from degradation (177–180). RAD52-mediated annealing of large regions of a homologous sequence, independent of RAD51-mediated strand invasion is key for the SSA (181). The N-terminal region of RAD52 is involved in the oligomeric ring formation leading to RAD52-ssDNA binding (182). The ring structure is crucial during different repair pathways by promoting annealing of complementary DNA strands. RAD52 also has a second DNA binding site that binds to dsDNA (183). Several studies demonstrated that unlike normal cells, RAD52 is required for the survival of cancer cells with loss-of-function mutation in genes such as BRCA1, BRCA2, PALB2, and RAD51 paralogs (184–186). Therefore, this differential effect facilitates RAD52 as a promising target to trigger synthetic lethality in BRCA-deficient tumor cells without affecting normal cells.

**Figure 11:**
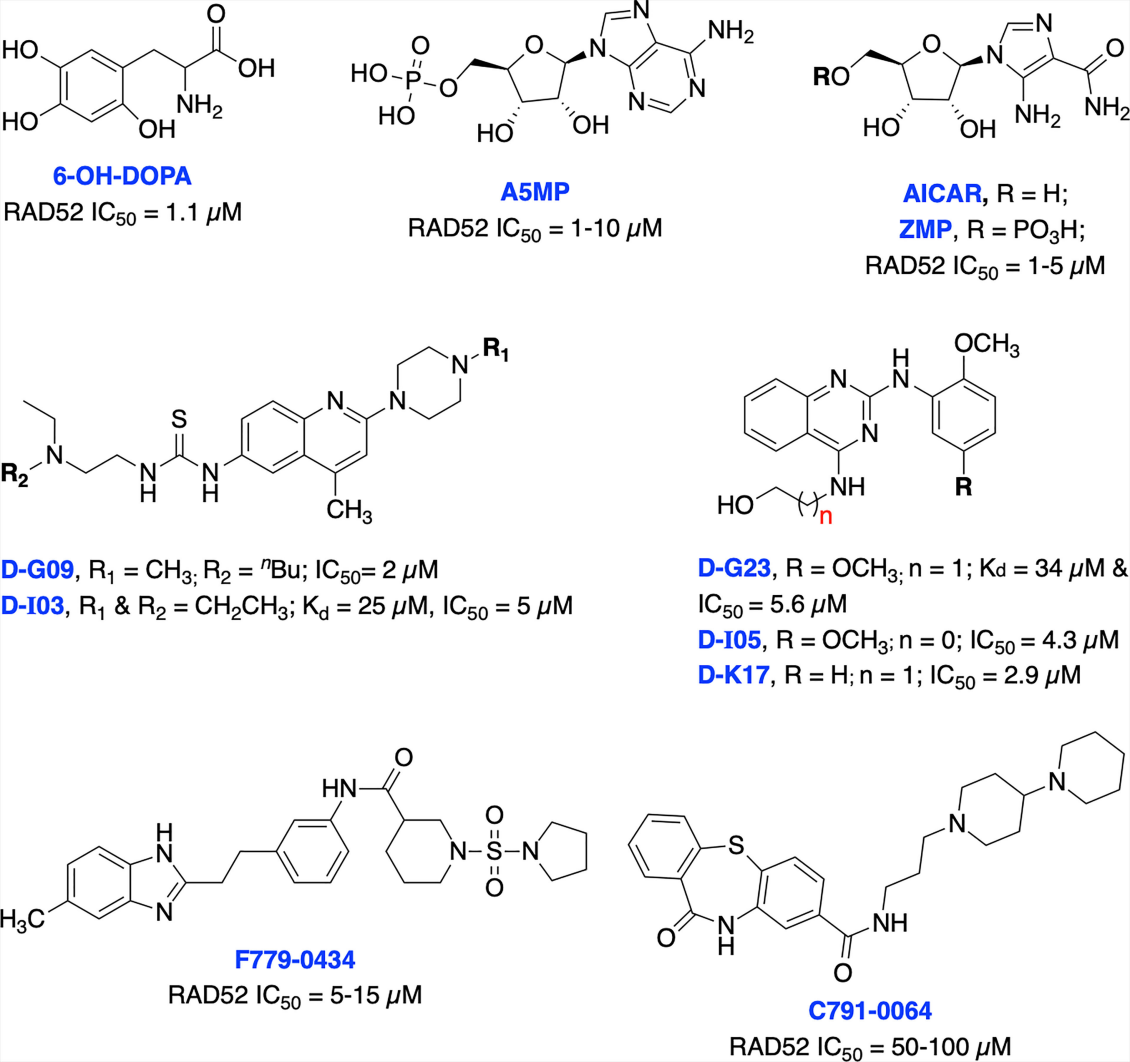
Small molecule inhibitors targeting RAD52 with their respective Kd/IC50 values for either RAD52 binding or ssDNA annealing activity.

To date, there have been several RAD52 inhibitors identified by various research groups (**Figure 11**) (179). Chandramouly et al. identified 6-OH-DOPA as a specific inhibitor to RAD52 ring structure formation through HTS. Notably, 6-OH-DOPA disrupts the heptamer and undecamer ring of truncated RAD52 (residues 1-209) into dimers (187), leading to abolished recruitment of RAD52 to ssDNA damage sites. 6-OH DOPA disrupted the association of ssDNA with RAD52 and consistently inhibited SSA in cells while having a minimal effect on HR and NHEJ in BRCA-proficient cells while increased level of apoptosis and DNA damage observed in BRCA1/2-deficient cells. In addition, 6-OH DOPA selectively halted proliferation of BRCA1/2 deficient TNBC cells, pancreatic cancer cells and patient-derived AML and CML cells. Another study reported Adenosine 5’-monophosphate (A5MP), its mimics 5-aminoimidazole-4-carboxamide ribonucleotide (AICAR) and 5’ phosphate (ZMP) as RAD52 inhibitors through virtual computer screening of FDA and NCI drug libraries (188). All three inhibitors inhibited RAD52-ssDNA binding, while cell permeable AICAR disrupted SSA repair and reduced cisplatin-induced RAD52-ssDNA foci formation in BRCA1-deficient leukemic cells. Both A5MP and AICAR exerted anti-tumor activity against BRCA-deficient cancer cells by triggering synthetic lethality. Huang et al. identified 17 putative inhibitors of RAD52 through HTS. Among these, D-G09 and D-I03 showed exquisite selectivity against RAD51 and anticancer activity in BRCA1/2 deficient pancreas, ovarian, and TNBC cells with no effect in BRCA1/2 proficient cells (189). Further biochemical studies confirmed that both inhibitors bind directly to RAD52, impairs its ssDNA-annealing activity and DNA pairing activity of RAD52 (D-loop formation) in the sub-micromolar range. D-I03 showed no significant effect on cisplatin-induced RAD51 foci formation although this compound significantly reduced level of SSA repair without influencing HDR indicating specific targeting of RAD52. In addition, structurally distinct compounds, D-G23, D-I05 and D-K17 also inhibited RAD52 ssDNA annealing, DNA pairing activities of RAD52 and preferentially inhibited at least two BRCA1/2-defficient cell lines.

Li et al. identified several RAD52 inhibitors through virtual HTS and docking studies with top compounds F779-0434 and C791-0064 inhibiting RAD52-ssDNA association and disrupting single strand annealing activity of RAD52, respectively and inducing synthetic lethality by suppressing the proliferation of BRCA2-deficient cancer cells at high concentrations (190, 191). Hengel et al. identified natural products (−)-epigallocatechin, epigallocatechin-3-monogallate and NP-004255 (RAD52 IC_50s_ = 1.8, 0.277 and 1.5 μM, respectively) as potent inhibitors of RAD52 by utilizing HTS and FRET-based assays. Both (−)-epigallocatechin and epigallocatechin-3-monogallate inhibited DSB repair and significantly reduced proliferation of BRCA2 and MUS81 deficient cells under conditions of replication stress (192).

While clearly in the developmental stages, each of the RAD52 inhibitors could offer potential for further development of effective treatment to improve therapeutic outcome of BRCA deficient malignancies in combination with PARPi.

### ERCC1-XPF Inhibitors

The structure-specific heterodimeric endonuclease ERCC1-XPF complex is primarily involved in NER but has roles in SSA and alt-NHEJ mediated DSB repair as well as interstrand cross-link (ICL) repair pathways due to its unique catalytic incision properties (193, 194). ERCC1 regulates DNA-protein and protein-protein interactions and is catalytically inactive while XPF which contains an inactive helicase-like motif, is involved in protein-protein interactions and DNA binding, and provides the endonuclease activity. The overexpression of ERCC1-XPF has been linked with poor responses to chemotherapy in various cancers including NSCLC, squamous cell carcinoma, ovarian cancer and melanoma while low ERCC1-XPF expression observed in testicular cancer has extended overall survival of cancer patients (195, 196). Further, ERCC1 deficient melanoma cells exhibited around 10-fold more sensitivity to cisplatin than ERCC1-proficient cells and in a xenograft mouse model as well (197). ERCC1-XPF became an interesting target to investigate in order to overcome resistance to chemotherapeutic agents due to its involvement in multiple key repair pathways.

The heterodimerization and localization of ERCC1 and XPF is required to constitute a functional and stable complex and essential for endonuclease activity. ERCC1-XPF interaction through their double helix–hairpin–helix (HhH2) domains is an essential requirement to stabilize ERCC1-XPF complex to promote catalytic activity (198, 199). Therefore, several research groups are targeting ERCC1-XPF HhH2 domain protein-protein interaction to develop novel inhibitors to increase sensitivity of existing therapies whose DNA-damaging effects are primarily repaired by ERCC1-XPF-dependent pathways.

**Figure 12:**
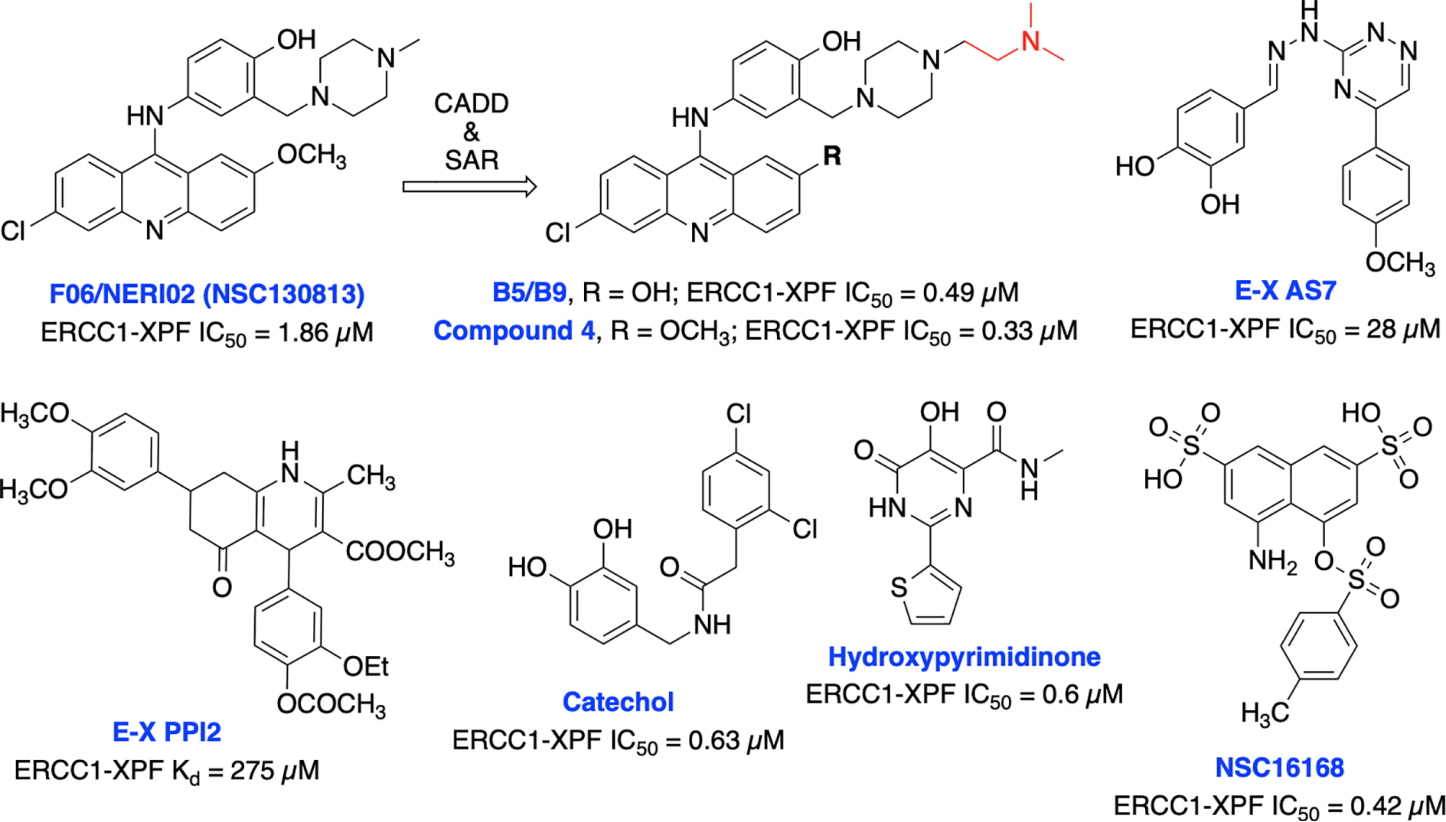
Small molecule inhibitors targeting ERCC1-XPF with their respective Kd/IC50 values for inhibition of ERCC1-XPF endonuclease activity.

Jordheim et al. identified F06/NERI02 (NSC130813) through *in silico* screening, as a small molecule inhibitor targeting ERCC1-XPF heterodimerization and demonstrated modest affinity for XPF and sensitized cancer cells to MMC and cisplatin (**Figure 12**) (200). In addition, F06 exhibited a synergy with PARPi olaparib in BRCA1-deficient breast cancer cells. However, suboptimal potency, toxicity and off-target effects of F06 restricted further biochemical and cellular studies (62). Recently, West and co-workers rationally modified the structure of F06 by utilizing computer-aided drug design (CADD) to identify potential binding interactions and further SAR studies to improve inhibition of ERCC1-XPF endonuclease activity. The lead compounds B5/B9 and compound 4 showed 3-fold improvement in inhibition activity compared to F06. The sensitivity to UV radiation and cyclophosphamide also increased significantly in reducing proliferation of metastatic colorectal cancer (201–203). Moreover, compound 4 showed lower lipophilicity and greater metabolic stability which makes this compound an interesting candidate for further advancement. McNeil et al. targeted three sites of the ERCC1-XPF HhH2 domain to identify possible inhibitors for the heterodimer by utilizing an *in silico* screening approach (204). They identified E–X AS7 which binds to ERCC1-XPF through a metal-based interaction, inhibits NER in low micromolar concentrations and specifically increases the cisplatin sensitivity of NER-proficient human and mouse cells. E-X PPI2 inhibited the NER activity in melanoma cells, showed marginal sensitivity to cisplatin treatment but caused significant reduction in the level of ERCC1-XPF heterodimer levels in ovarian cancer cells. However, the medium-high micromolar range binding affinity (Kd) and inhibitory potency (IC_50_) makes these compounds unsuitable for further studies. A series of highly potent and selective catechols, hydroxylimides/hydroxy pyrimidinones have been identified as ERCC1-XPF inhibitors through *in silico* HTS and SAR approach (205, 206). Most of the compounds from these series showed good selectivity for ERCC1-XPF against FEN-1 and DNase I; however, potential *in vitro* and *in vivo* effects of these compounds are not documented yet. Patrick and co-workers targeted the active site on the XPF nuclease domain and identified NSC16168 as a potent ERCC1-XPF inhibitor by performing a HTS using the NCI-DTP (National Cancer Institute Developmental Therapeutics Program) diversity database. NSC16168 significantly enhanced cisplatin antitumor activity in a lung cancer xenograft model (207).

Overall efforts resulted in several potent ERCC1-XPF endonuclease inhibitors which are capable to diminish NER activity and enhance the cytotoxicity of platinum-based chemotherapeutics although these inhibitors are not explored in targeting DSB repair and its defects for cancer therapy. Moreover, the lack of structural insights, selectivity against other endonucleases and most importantly limited utilization of these inhibitors in targeting DSB repair restricts their further advancement into the clinic.

### DNA Polymerase Theta (Pol θ) Inhibitors

Pol θ (*gene* name, *PolQ*) belongs to the error-prone A family of DNA polymerases and is a critical component of the alt-NHEJ (MMEJ or TMEJ) repair pathway of resected DSBs. Biochemical and mechanistic studies have shown that the helicase domain of Pol θ displaces RPA bound to the ssDNA overhang and facilitates joining of short microhomologies to the two DNA strands that flank a DSB. The polymerase domain of Pol θ initiates DNA synthesis to fill in the DNA gaps, prior to the ligation step employed by DNA Ligase I or III. In addition, Pol θ also plays an important role in joining unprotected telomeres in alt-NHEJ pathway (14, 208–211). Alt-NHEJ serves as an essential backup pathway to repair DSBs when HDR and NHEJ pathways are compromised in cancer cells such as germline *BRCA*-gene deficient cancer cells. Recently, Pol θ emerged as a new promising drug target to trigger the synthetic lethality between loss of the PolQ gene and deficiencies in DSB DNA repair-related tumor suppressor genes including BRCA1/2, ATM and FANCD2 for the treatment of HDR-deficient tumors (212–214). The expression of Pol θ is particularly high in subtypes of breast and ovarian cancers featuring loss of HDR activity and Pol θ-depletion reduced the survival of HR-deficient cancer cells in the presence of PARPi, cisplatin, or MMC (214). Pol θ overexpression also found in other cancers, including stomach, lung and colon (215). In addition, the higher expression of Pol θ is correlated with shorter relapse-free survival compared to patients with relatively lower expression of Pol θ. Feng et al. employed CRISPR-based genetic screening and identified 140 genes that are synthetically lethal with Pol θ, highlighting the impact of Pol θ inhibitor for cancer therapy (216).

**Figure 13:**
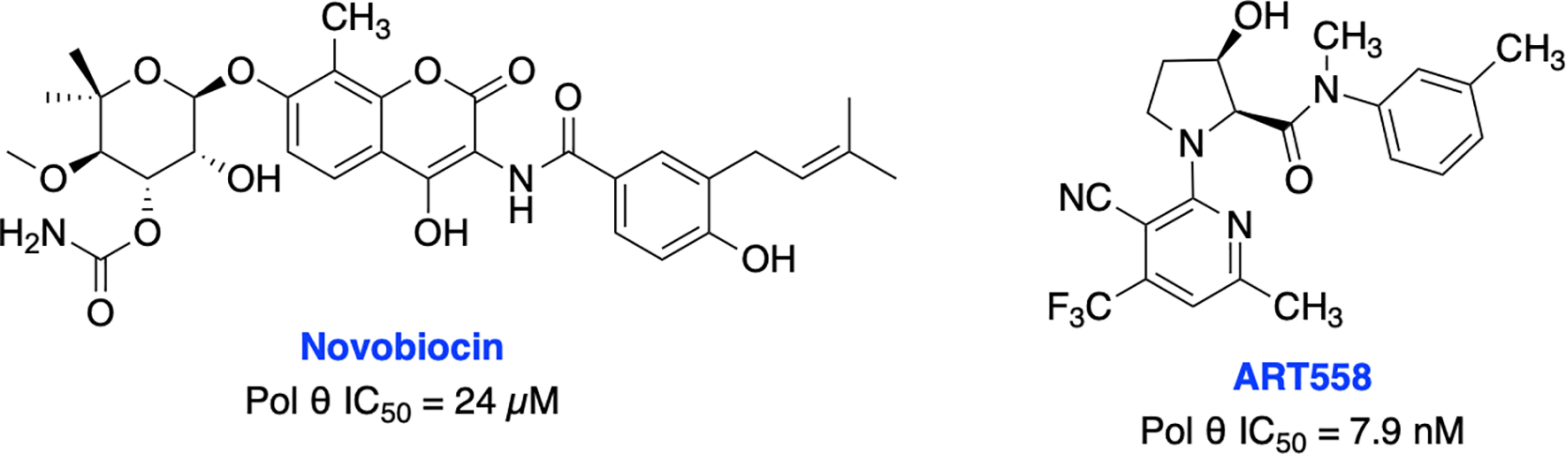
Small molecule inhibitors targeting Pol θ with their respective IC50 values for inhibition of polymerase activity.

Recently, Zhou et al. identified antibiotic novobiocin (NVB) as a specific potent inhibitor of human Pol θ (**Figure 13**) which inhibited alt-NHEJ repair and selectively killed HDR-deficient (both BRCA1- and BRCA2-deficient) cells over wild-type cells and significantly enhanced the cytotoxic effect of PARPi in HDR-deficient tumor cells in cellular as well as in xenograft and PDX mouse models (217). Most importantly NVB also killed HDR-deficient, PARPi-resistant tumor cells. Artios Pharma in collaboration with the Institute of Cancer Research (UK) identified ART558 as a highly potent and specific small molecule Pol θ inhibitor (213). ART558 exhibited not only *BRCA*-gene synthetic lethality, but also targets cells with PARPi resistance caused by defects in 53BP1/Shieldin DNA repair complex. There is a possibility that Pol θ inhibitors might be a more suitable treatment option than PARPi for combination with existing DNA-damaging chemotherapies. Several biopharmaceutical companies are currently pursuing Pol θ as a therapeutic target and the first orally bioavailable Pol θ inhibitor ART4215 (structure is not disclosed) is currently in Phase 1/2 clinical trials where it is being investigated as a monotherapy and in combination with PARPi talazoparib in patients with advanced or metastatic solid tumors (NCT04991480, https://clinicaltrials.gov/ct2/show/NCT04991480).

### RecQ and MCM Helicases Inhibitors

**Figure 14:**
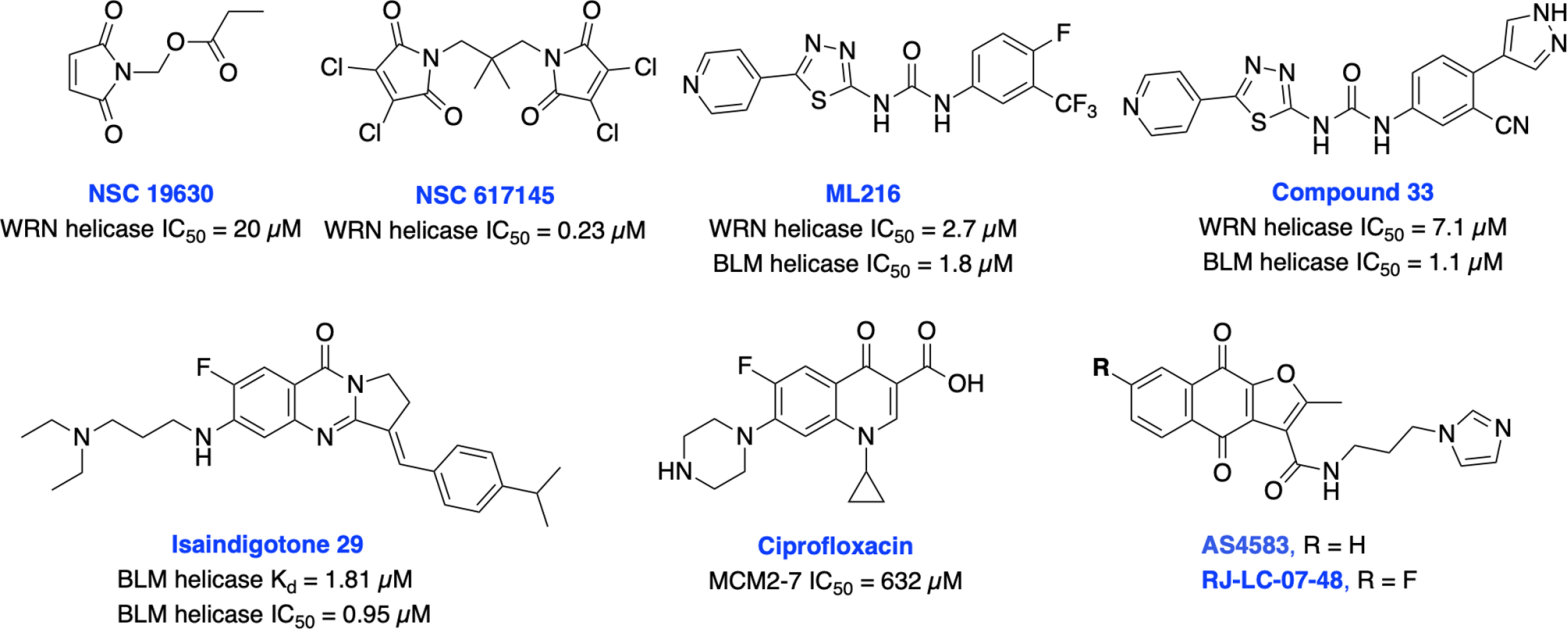
Small molecule inhibitors targeting WRN, BLM and MCM helicases with their respective IC_50_ values.

It is well established that RecQ helicases play an important role in DSB repair and the maintenance of genome stability. However, a direct or passive role of each RecQ helicase’s enzymatic activity in NHEJ, HDR, TMEJ and SSA mediated DSB repair pathway is yet to be elucidated (43). RecQ proteins are highly conserved from bacteria to humans, and the reduced RecQ helicases activity is associated with cancer predisposition, metastasis and premature aging. In contrast, overexpression of RecQ helicases may promote carcinogenesis and RecQ helicases are highly upregulated in various cancers (218, 219). Aggarwal et al. identified NSC 19630 and NSC 617145 (**Figure 14**) as WRN inhibitors through HTS of the National Cancer Institute (NCI) diversity set of compounds. Both NSC compounds dramatically impaired growth and proliferation, induced apoptosis in a WRN-dependent manner, and DSBs and chromosomal abnormalities in cellular models (220, 221). However, the presence of the maleimide group in both compounds may restrict their further development due to its propensity for non-specific covalent interactions. The same group recently identified several non-specific reversible and irreversible helicase inhibitors through HTS using a larger library of approximately 350,000 small molecules (222). Several studies identified WRN synthetic lethal vulnerability in cancers with microsatellite instability (218, 223, 224), suggesting specific WRN inhibitors hold great potential to target microsatellite instability tumors to enable a clear stratification path in the clinic.

BLM helicase plays a multifaceted role in HDR pathway as it is required for the early phase of the pathway to stimulate resection of DSB ends or displacement of the invading strand of DNA displacement loops as well as at the terminal steps in dissolution of double Holliday junctions (43). Nguyen et al. identified the first BLM inhibitor by utilizing HTS and further structural optimization efforts yielding ML216 and compound 33 as potent inhibitors of the DNA unwinding activity of both BLM and WRN (225, 226). ML216 exhibited cellular induction of sister chromatid exchanges and demonstrated selective antiproliferative activity in BLM-positive cells but not those lacking BLM. However, further preclinical studies may be restricted due to poor selectivity, solubility, and cell permeability of these inhibitors. Recently, Yin et al. identified isaindigotone derivatives as novel BLM helicase inhibitors that disrupted the recruitment of BLM at DNA DSB sites. BLM inhibition by their lead compound promoted accumulation of RAD51, regulated HDR repair, and synergized cytotoxicity of cisplatin and the RAD51 inhibitor, RI-1 (227). BLM and other helicases are attractive targets for the development of cancer therapeutics which rely on synthetic lethality effects for targeting tumors with preexisting DNA repair deficiencies. Minichromosome maintenance (MCM) complex is a family of six proteins 2-7 (MCM2-7) that are activated by forming a holo-helicase CMG complex with Cdc45 and the hetero-tetrameric GINS complex (Cdc45-MCM2-7-GINS). CMG complex is cell-cycle regulated and responsible for unwinding DNA forks during DNA replication. MCM2-7 proteins have essential roles in DNA replication particularly under replicative stress where they activate dormant replication origins which allows for continued genome replication in spite of replication stress. Increased levels of MCM2-7 protein expression have been observed in a variety of cancers (39, 42). Initially, Simon et al. identified ciprofloxacin which preferentially inhibits MCM2-7 at higher concentrations than its normal therapeutic range (228). However, most recently inhibition of MCM2-7 activity by ciprofloxacin significantly delayed neuroendocrine prostate cancer (NEPC) cell growth and migration *in vitro*, exhibited potent anti-tumor effects in an NEPC xenograft model, and partially reversed neuroendocrine features (229). Alshahrani et al. identified UEFS99, UEFS137 and UEFS428 as MCM7 inhibitors from the natural compounds databases using *in silico* computational screening, however further *in vitro* and *in vivo* studies are needed to validate target engagement (230). A furanonaphthoquinone-based small molecule, AS4583 was identified as an MCM2 inhibitor through phenotypic screening and target deconvolution (231). Further mechanistic studies revealed that AS4583 inhibited cell-cycle progression and reduced DNA replication by inducing proteasomal degradation of MCM complex which ultimately contributed to the death of NSCLC cells. Subsequently, structural optimization of AS4583 led to compound RJ-LC-07-48 which showed greater potency in drug-resistant NSCLC cells and in mice bearing H1975 tumor xenografts. Overall, MCM complex can serve as a potential target for cancer therapy. Further exploration of design, screening and medicinal chemistry efforts are needed to develop MCM2-7 complex-specific inhibitors for better clinical outcomes.

## DSB Repair Inhibitors for Combination Therapy, Induction of Synthetic Lethality and Precision Genome Editing

While there remain no FDA approved inhibitors of non-PIKKs within the DSB repair pathways, the future applications of such compounds may include use within combination chemotherapy regimens, as chemo- or radiosensitizers, induction of synthetic lethality in HDR-deficient cancer subtypes, and as an adjuvant therapy in precision genome editing. As described in the above corresponding sections, each of the non-PIKK pharmacological targets, whether involve directly or indirectly mediate repair of DSBs induced by either DNA damaging agents or IR, and combination therapy with either DNA damaging agents or IR has been the natural step towards maximizing synergistic efficacy, overcoming resistance, and reducing the toxicities associated with existing chemo- and radiotherapy. Provided that the preponderance of cancer patients receives DNA-damaging drugs or IR and later experience disease progression, the therapeutic potential for agents that augment the response to such therapies is large.

Synthetic lethality refers to any scenario whereby loss of two gene products produces cellular death, but loss of either individually is non-lethal. In the setting of cancer treatment, synthetic lethality is a term usually used in reference to disruption of the repair of DNA nicks in HDR-deficient cancers which yields DSBs that are repaired by error-prone pathways resulting in cell death or senescence (232). The clinically available PARP inhibitors (PARPis) olaparib, rucaparib, talazoparib, and niraparib operate by this mechanism and are frequently employed in cancers where HDR-deficiency is conferred by BRCA mutations. PARPis remain the sole class of approved anticancer drugs capable of exploiting this unique vulnerability. However, more than 40% patients with BRCA mutations fail to respond to PARPis and resistance mechanisms have been described indicating new classes of medications capable of inducing synthetic lethality are needed (233). Two particularly noteworthy non-PIKKs targets within the DSB repair pathways whose inhibition have been probed for synthetic lethality in the setting of PARPi resistance include DNA polymerase θ and RAD52. The activity of DNA polymerase θ offers an escape pathway beyond NHEJ *via* alt-NHEJ in the setting of BRCA mutations (70, 208). An siRNA knockdown of DNA polymerase θ produces synthetic lethality in BRCA2 mutation variants (214) and the aforementioned DNA polymerase θ inhibitor ART558 retains preclinical efficacy even in the presence of 53bp1 mutations which are known to confer PARPi resistance (213). The RAD52 deficiency leads to loss of a compensatory DNA repair pathway resulting in genomic instability and persistent cell death in BRCA1/2-deficient cells. Intriguingly, RAD52 is thought to be capable of orchestrating HDR even in BRCA1/2 mutants and thus may also play a role in PARPi resistance. Targeting RAD52 for the induction of synthetic lethality could potentially improve the therapeutic outcome of BRCA-deficient malignancies treated with PARPi and restrict the emergence of drug-induced toxicity to normal tissues (179, 208).

CRISPR-Cas9 genome editing offers the possibility to prevent, treat, or even cure human diseases that are initiated by or maintained by genetic aberrations (234). Notwithstanding other barriers to the clinical application of CRISPR-Cas9 for genome editing such as selective delivery and the requirement for protospacer adjacent motifs (PAM) at the targeted region of a locus, a major challenge this platform faces is management of the DSBs created both on-target and off-target (235, 236). Provided that HDR is only available in the G2/S phases of the cell cycle because of the requirement for a sister chromatid, the predominant NHEJ pathway must be regulated in precision genome editing to preempt chromosomal rearrangements and large indels. DNA DSB repair inhibitors could help in enhancing precision genome editing as well as improving the safety of gene targeting. NHEJ inhibitors and HDR modulators can be exploited to increase the current efficiency of nuclease-based HDR mediated gene editing alongside CRISPR towards the more precise HDR mediated repair while decreasing inaccurate integration events. However, specificity, efficacy and toxicity associated with DSB repair inhibitors targeting NHEJ pathway restricted utilization of these inhibitors in CRISPR-Cas9 genome editing (89, 103, 104). Future availability of an arsenal of DSB-repair inhibitors capable of directing DSB repair by HDR will foster the arrival of precision genome editing within clinical practice.

We have summarized a list of targeted proteins and their respective inhibitors, mechanism of their action, binding affinity or *in vitro* potency, indication along with cellular activity and their phase of development in **Table 1**.

**Table 1:** A summary of non-PIKKs DSB Repair inhibitors.

Targeted Protein and Inhibitors	Mechanism of Action and *In vitro* potency	Cellular Activity	Phase of Development
**Ku70/80**			
STL127705 (Compound L)	• Disrupts Ku-DNA binding activity and inhibits DNA-PK enzymatic activity. Ku IC_50_ = 3.5 μM DNA-PK IC_50_ = 2.5 μM	• Single agent activity and radiosensitivity in glioblastoma and prostate epithelial cancer cells. IC_50_ = 20-35 μM	Pre-Clinical
5102	• Disrupts Ku-DNA binding activity and inhibits DNA-PK enzymatic activity. Ku IC_50_ = ~3.0 μM DNA-PK IC_50_ = ~0.3 μM	NR	Pre-Clinical
5135	• Disrupts Ku-DNA binding activity and inhibits DNA-PK enzymatic activity. Ku IC_50_ = ~2.5 μM DNA-PK IC_50_ = ~0.1 μM	NR	Pre-Clinical
68	• Disrupts Ku-DNA binding activity and inhibits DNA-PK enzymatic activity. Ku IC_50_ = 6.02 μM DNA-PK IC_50_ = 3.1 μM	• Inhibits cellular NHEJ activity. • Potentiates the cellular activity of bleomycin.	Pre-Clinical
149	• Disrupts Ku-DNA binding activity and inhibits DNA-PK enzymatic activity. Inhibits *in vitro* NHEJ. Ku IC_50_ = 3.72 μM DNA-PK IC_50_ = 0.5 μM	• Inhibits cellular NHEJ activity.	Pre-Clinical
322	• Disrupts Ku-DNA binding activity and inhibits DNA-PK enzymatic activity. Ku IC_50_ = 2.66 μM DNA-PK IC_50_ = 0.11 μM	• Inhibits cellular NHEJ activity. • Potentiates the cellular activity of etoposide and IR in lung cancer cells.	Pre-Clinical
245	• Disrupts Ku-DNA binding activity and inhibits DNA-PK enzymatic activity. Ku IC_50_ = 1.99 μM DNA-PK IC_50_ = 0.24 μM	• Inhibits cellular NHEJ activity. • Potentiate the cellular activity of bleomycin and IR in lung cancer cells. • Shows modulation of CRISPR/cas9 mediated gene insertion.	Pre-Clinical
**Artemis**			
Ebselen	• Interacts with zinc finger motif of Artemis and inhibit its endonuclease activity IC_50_ = 8.5 μM	NR	Pre-Clinical
Disulfiram	• Interacts with zinc finger motif of Artemis and inhibit its endonuclease activity IC_50_ = 10.8 μM	NR	Pre-Clinical
Auranofin	• Interacts with zinc finger motif of Artemis and inhibit its endonuclease activity IC_50_ = 46 μM	NR	Pre-Clinical
Ceftriaxone	• Interacts with zinc finger motif of Artemis and inhibit its endonuclease activity IC_50_ = 65 μM	NR	Pre-Clinical
**DNA Ligase IV**			
L189	• Binds in DNA-binding pocket of the DBD. • Inhibits DNA ligases I, III, and IV in DNA joining assay. Ligase I IC_50_ = 5 μM, Ligase III IC_50_ = 9 μM, Ligase IV IC_50_ = 5 μM	• Single agent activity and radiosensitivity in colon and breast cancer cells. IC_50_ = 20-35 μM	Pre-Clinical
SCR7-cyclized and SCR7-pyrazine	• Inhibit Ligase IV-mediated end joining and V(D)J recombination. • Blocks NHEJ in a Ligase IV-dependent manner. SCR7-cyclized K_d_ = 2.35 μM SCR7-pyrazine K_d_ = 0.5 μM	• Single agent activity in leukemic, cervical, breast cancer cells and radiosensitivity in cervical cancer cells. IC_50_ = 50-250 μM	Pre-Clinical
SCR130	• Inhibits Ligase IV-mediated end joining in concentration dependent manner Ligase IV IC^50^ = NR	• Single agent activity and radiosensitivity in leukemic and cervical cancer cells. IC_50_ = 2-14 μM	Pre-Clinical
**MRE11**			
Mirin	• Binds in the active site of MRE11 and blocks DNA phosphate backbone rotation which selectively blocks Mre11 exonuclease activity. • Inhibits the MRN-dependent activation of ATM without affecting its kinase activity (IC_50_ = 66 μM). MRE11 IC_50_ = ~200 μM	• Abolishes the G2/M checkpoint and HDR DNA repair in human cells. • Inhibits dsDNA end resection in A549 cells. • Single agent activity in neuroblastoma, glioblastoma, prostate cancer cells and chemosensitivity to DNA damaging agents in glioblastoma cells. IC_50_ = 15-72 μM	Pre-Clinical
PFM01 and PFM03	• Binds near the dimer interface by blocking ssDNA-binding and selectively blocks Mre11 endonuclease activity. MRE11 IC_50_ = ~75-100 μM	• Prevents dsDNA end resection in A549 cells (IC_50_ = 50-75 μM).	Pre-Clinical
PFM39	• Binds in the active site similar to Mirin and selectively blocks Mre11 exonuclease activity MRE11 IC_50_ = < 100 μM	• Prevents dsDNA end resection in A549 cells (IC_50_ = 50-75 μM).	Pre-Clinical
**RPA**			
NSC15520 (FPA)	• Disrupts RPA DBD-F (N-terminal RPA70N) interactions with Rad9 and p53. • Inhibits RPA dsDNA binding, and helix destabilization activity without affecting ssDNA binding activity. RPA IC_50_ = 10 μM	NR	Pre-Clinical
HAMNO	• Disrupts RPA DBD-F (N-terminal RPA70N) interactions with Rad9 • Prevents DBD-F-dependent unwinding of DNA by RPA but does not prevent RPA ssDNA binding RPA IC_50_ = >50 μM	• Single agent activity in head and neck and glioblastoma cancer cells, sensitizes head and neck cancer cells to etoposide and glioblastoma cancer stem-like cells to IR. IC_50_ = 5-33 μM	Pre-Clinical
VU079104	• Binds in basic cleft of N-terminal RPA70N • Inhibits the interaction of RPA70N with the peptide binding motif derived from ATRIP RPA K_d_ = 41 μM	NR	Pre-Clinical
Anthranilic acid- based inhibitors	• Binds to N-terminal RPA70N RPA K_d_ = 0.81 μM	NR	Pre-Clinical
Chlorobenzothio-phene-and Pyrazole-based inhibitors	• Binds in basic cleft of N-terminal RPA70N and displaces the binding of an ATRIP-derived peptide to RPA. RPA K_d_ = 0.19-18 μM	NR	Pre-Clinical
MCI13E and MCI13F (Irreversible inhibitors)	• Covalently binds with DBD A and B of RPA. RPA IC_50_ = 10-16 μM	• Single agent activity in lung and ovarian cancer cells and synergism with cisplatin in lung cancer cells. IC_50_ = 1-5 μM	Pre-Clinical
TDRL-505 and TDRL-551	• Inhibits DNA-binding activity of RPA targeting DBD-A and DBD-B in the 70-kDa subunit of RPA RPA IC_50_ = 18-38 μM	• Single agent activity in lung and ovarian cancer cells and synergism with cisplatin and etoposide in lung cancer cells and xenograft model. IC_50_ = 25-30 μM	Pre-Clinical
43/NERx-329 and 44/NERx-2004	• Inhibits DNA-binding activity of RPA targeting DBD-A and DBD-B in the 70-kDa subunit of RPA RPA IC_50_ = 4.9-10 μM	• 43/NERx-329 shows degradation of replication forks upon stalling and RPA exhaustion, single agent activity in a broad spectrum of cancer cells and synergism with cisplatin, etoposide, bleomycin, BMN673, NU7441 and VE821 in lung cancer cells. IC_50_ = 3-10 μM	Pre-Clinical
**RAD51**			
DIDS	• Directly binds to RAD51 and inhibits both RAD51-ssDNA and RAD51-dsDNA binding. • Inhibits the RAD51-mediated strand exchange and homologous pairing in the absence of RPA. RAD51 K_d_ = 2 μM	NR	Pre-Clinical
Halenaquinone	• Specifically inhibits the RAD51-dsDNA binding. RAD51 IC_50_ = 30-60 μM	NR	Pre-Clinical
RI-1 (Irreversible inhibitor)	• Inhibits RAD51 binding to ssDNA by covalently modifying C319 thiol group of RAD51 • Inhibits D-loop formation of RAD51. IC_50_ = 6.82 μM	• Inhibits HR DNA repair and disrupts DNA damage induced RAD51 foci formation. • Sensitizes osteosarcoma, cervical, and breast cancer cells to MMC by triggering synthetic lethality. IC_50_ = 20-40 μM	Pre-Clinical
RI-2	• Reversibly Inhibits RAD51 binding to ssDNA. IC_50_ = 44.17 μM	• Inhibits HR DNA repair and sensitizes HEK293 cells to MMC by triggering synthetic lethality. LD_50_ = 70 μM	Pre-Clinical
RS-1	• Enhances binding of RAD51 to ssDNA and dsDNA. • Enhances recombination activities of RAD51 by locking its active conformation, without affecting ATP hydrolysis. RAD51 K_d_ = 107 nM	• Enhances HR activity, D-loop formation and the formation of toxic RAD51 complexes on undamaged chromatin. • Leads to the accumulation of RAD51 foci in prostate cancer cells but not in normal cells which is independent of DNA damage. • Enhances cellular resistance to cisplatin at ~7.5 μM.	Pre-Clinical
B02	• Specifically binds to RAD51 and disrupts binding of dsDNA to RAD51-ssDNA Filament. RAD51 IC_50_ = 27.4 μM	• Inhibits DSB-induced HR DNA repair and RAD51 foci formation induced by DNA damage. • Enhances sensitivity of cancer cells to IR, MMC, cisplatin, etoposide and topotecan. • Significantly increases sensitivity of doxorubicin in myeloma cells and MMS in combination with PARPi in MEF cells by triggering synthetic lethality.	Pre-Clinical
B02-iso and *p*-I-B02-iso	• Binds within the dimerization interface of a RAD51 filament. B02-iso RAD51 K_d_ = 14.6 μM *p*-I-B02-iso RAD51 K_d_ = 1.4 μM	• Inhibits HR DNA repair and RAD51 foci formation in cancer cells induced by DNA damage. • Single agent activity in TNBC cells and enhances the sensitivity of BRCA-proficient TNBC cells to the PARPi, olaparib through synthetic lethality. • Enhances radiosensitivity in combination with olaparib in different cancer cells by inducing synthetic lethality. IC_50_ = 2.6-11.9 μM	Pre-Clinical
IBR2	• Directly binds to RAD51, disrupts the RAD51-BRCA interaction and RAD51 multimerization. RAD_51_ IC_50_ = 10 μM	• Specifically inhibits RAD51-mediated HR, diminishes IR-induced RAD51 foci and enhances proteasomal degradation of RAD51. • Single agent activity and enhances chemosensitivity to receptor tyrosine kinase and microtubule inhibitors in a broad spectrum of cancer cells by inducing synthetic lethality. • Overcomes CML drug resistance. IC_50_ = 12-16 μM	Pre-Clinical
IBR120	• Directly binds to RAD51, disrupts the RAD51-BRCA interaction and RAD51 multimerization. RAD_51_ IC_50_ = 3-10 μM	• Inhibits HR DNA repair and single agent activity in a broad spectrum of cancer cells. IC_50_ = 3-9.5 μM	Pre-Clinical
Triazole-based inhibitors	• Disrupts the RAD51-BRCA2 interaction and mimics the effect of BRCA2 mutation. RAD51 IC_50_ = 8-53 μM	• Inhibits HR DNA repair and increases the formation of DSBs in combination with olaparib. • Enhances the sensitivity of pancreatic cancer cells to olaparib by inducing synthetic lethality to the functional BRCA2. IC_50_ = 20-30 μM	Pre-Clinical
Dihydroquinolone pyrazoline (DHQP)	• Disrupts the RAD51-BRCA2 interaction and mimics the effect of BRCA2 mutation. RAD51 IC_50_ = 19 μM	• Inhibits HR DNA repair, reduces RAD51 foci formation induced by DNA damage. and synergizes with olaparib in pancreatic cancer cells to trigger synthetic lethality. IC_50_ = 20-30 μM	Pre-Clinical
CYT01B and CYT-0851	• Directly binds to RAD51 and disrupts RAD51 focus formation which reduces the nuclear concentration of RAD51 and promotes RAD51 protein degradation.	• Inhibits HR activity and anticancer activity in cells expressing activation-induced cytidine deaminase (AID), a protein that promotes formation of DSBs. • Shows synergy with cisplatin, PARP and ATR inhibitors in various cancer cells by inducing synthetic lethality. IC_50_ = 20 nM-5 μM	**CYT-0851** in phase 1/2 clinical trials for hematologic malignancies and advanced solid tumors. (NCT03997968)
**RAD52**			
6-OH-DOPA	• Disrupts the association of ssDNA with RAD52 and RAD52 oligomers. RAD52 IC_50_ = 1.1 μM	• Inhibits RAD52 foci induced by cisplatin and inhibits SSA with minimal effect on HR and NHEJ in BRCA-proficient cells. • Single agent activity in BRCA1/2 deficient TNBC cells, pancreatic cancer cells and patient-derived AML and CML cells through synthetic lethality. IC_50_ = 5-75 μM	Pre-Clinical
A5MP, AICAR and AICAR 5’-phosphate (ZMP)	• Disrupts the RAD52-ssDNA interaction A5MP RAD52 IC_50_ = 1-10 μM AICAR & ZMP RAD52 IC_50_ = 1-5 μM	• AICAR reduces RAD52 foci formation and inhibits SSA activity. • AICAR reduces growth of BRCA1-mutated breast and BRCA2-mutated pancreatic cancer cells by inducing synthetic lethality. IC_50_ = 2-20 μM	Pre-Clinical
D-G09 and D-I03	• D-G09 and D-I03 bind directly to RAD52, impairs RAD52 ssDNA-annealing activity (IC_50_ = 2 and 5 μM, respectively) and DNA pairing activity (D-loop formation) with IC_50_ = 14 and 8 μM, respectively.	• D-I03 significantly reduces level of SSA repair without influencing HDR and shows no effect on cisplatin-induced RAD51 foci formation. • D-G09 and D-I03 shows anticancer activity in BRCA1/2 deficient leukemic, pancreas, ovarian, and TNBC cells by inducing synthetic lethality. IC_50_ = 2.5-16 μM	Pre-Clinical
D-G23, D-I05 and D-K17	• Bind directly to RAD52, impairs RAD52 ssDNA-annealing activity (IC_50_ = 2.9-5.6 μM) and DNA pairing activity (D-loop formation) with IC_50_ = 4.8-7.2 μM.	• Shows anticancer activity in BRCA1/2-defficient cancer cells through synthetic lethality. IC_50_ = 9-26 μM	Pre-Clinical
F779-0434 and C791-0064	• F779-0434 inhibits RAD52-ssDNA association (IC_50_ = 5-15 µM) and C791-0064 disrupting single strand annealing activity of RAD52 (IC_50_ = 50-100 µM).	• Shows anticancer activity in BRCA1/2-defficient pancreatic cancer cells through synthetic lethality. IC_50_ = 5-80 μM	Pre-Clinical
**ERCC1-XPF**			
F06/NERI02 (NSC130813)	• Interacts with the XPF double helix−hairpin−helix (HhH2) domain to disrupt ERCC1-XPF heterodimerization. • Inhibits ERCC1-XPF endonuclease activity. ERCC1-XPF IC_50_ = 1.86 µM	• Inhibits the interaction between XPF and ERCC1 in lung cancer cells. • Single agent activity and chemosensitivity to MMC and cisplatin in lung and colorectal cancer cells and radiosensitivity in lung cancer cells. • Shows synergy in BRCA1-defficient breast cancer cells by inducing synthetic lethality. IC_50_ = 0.79-3 μM	Pre-Clinical
B5/B9 and Compound 4	• Binds in the subunit interaction domain of ERCC1−XPF. • Inhibits ERCC1-XPF endonuclease activity. • B5/B9 ERCC1-XPF IC_50_ = 0.49 µM Compound 4 ERCC1-XPF IC_50_ = 0.33 µM	• Both compounds inhibit the removal of bulky DNA lesions, such as cyclobutane pyrimidine dimers (CPDs) in UV-irradiated cells. • Both compounds enhance the sensitivity of colorectal cancer cells to UV radiation and cyclophosphamide. B5/B9 IC_50_ = ~17 µM Compound 4 IC_50_ = 3.5-6 µM	Pre-Clinical
E-X AS7 and E-X PPI2	• Interacts with the XPF double helix−hairpin−helix (HhH2) domain to disrupt ERCC1-XPF heterodimerization. • Inhibits ERCC1-XPF endonuclease activity. E-X AS7 ERCC1-XPF IC_50_ = 28 µM E-X PPI2 ERCC1-XPF K_d_ = 275 µM	• Inhibit NER and enhance the sensitivity of NER-proficient melanoma cells to cisplatin. • E-X PPI2 reduces ERCC1-XPF heterodimer levels in ovarian cancer cells. E-X PPI2 IC_50_ = 20 µM	Pre-Clinical
Catechol and Hydroxy- pyrimidinone	• Inhibit ERCC1-XPF endonuclease activity and show selectivity for ERCC1-XPF against FEN-1 and DNase. ERCC1-XPF IC_50_ = 0.6 µM	• Catechol inhibits NER activity and enhances the sensitivity of melanoma cells to cisplatin.	Pre-Clinical
NSC16168	• Inhibits ERCC1-XPF endonuclease activity and DNA binding ability of ERCC1-XPF. ERCC1-XPF IC_50_ = 0.42 µM	• Potentiates cisplatin efficacy in lung cancer cells and xenograft model.	Pre-Clinical
**Pol θ**			
Novobiocin	• Binds to the Pol θ ATPase domain and inhibits its ATPase activity. Pol θ IC_50_ = 24 µM	• Inhibits the TMEJ activity in cells and induces excessive DSB end resection and RAD51 foci. • Inhibits HDR-deficient (BRCA1- and BRCA2) breast and ovarian tumors in GEMM, xenograft and PDX models. • Enhances the cytotoxic effect of PARPi in HDR-deficient tumor cells, xenograft and PDX models and overcomes acquired PARPi resistance in HR-deficient ovarian cancer PDX model by triggering synthetic lethality. IC_50_ = 25-50 µM	Pre-Clinical
ART558 and ART4215	• Inhibit Pol θ polymerase activity and Pol θ-mediated DNA DSB repair. ART558 Pol θ IC_50_ = 7.9 nM	• ART558 elicits DNA damage and synthetic lethality in BRCA1- or BRCA2- deficient cancer cells, xenograft model and enhances the effects of a PARPi in BRCA deficient cancer cells. • Induces synthetic lethality in PARPi resistance cells with defects in the Shieldin complex. IC_50_ = 0.5-1.5 µM	**ART4215** in phase 1/2 clinical trials as a monotherapy and in combination with PARPi, talazoparib for advanced or metastatic solid tumors. (NCT04991480)
**RecQ and MCM** **helicases**			
NSC 19630 and NSC 617145	• Inhibit WRN helicase activity but not its nuclease activity. NSC 19630 IC_50_ = 20 µM NSC 617145IC_50_ = 0.23 µM	• Both compounds show single agent activity and accumulation of DSBs and formation of stalled replication forks. • NSC 19630 sensitizes cells to G-quadruplex-binding compound telomestatin, or PARP inhibitor by inducing synthetic lethality. • NSC 617145 induces WRN binding to chromatin and proteasomal degradation, enhances Fanconi Anemia (FA) mutated cells activity to MMC and activates ATM by inducing synthetic lethality. IC_50_ = 2-5 µM	Pre-Clinical
ML216 and Compound 33	• Inhibit helicase activity, DNA unwinding activity of both BLM and WRN and disrupt the DNA binding activity of BLM. ML216 WRN IC50 = 2.7 µM and BLM IC_50 _= 1.8 µM. Compound 33 WRN IC_50_ = 7.1 µM and BLM IC_50_ = 1.1 µM	• ML216 enhances sister chromatid exchange, single agent activity and sensitivity to aphidicolin in BLM expressing cells.	Pre-Clinical
Isaindigotone 29	• Inhibits BLM helicase activity and disrupts the recruitment of BLM at DNA DSB sites. BLM IC_50_ = 0.95 µM	• Induces DNA damage, promotes the accumulation of RAD51 at DNA DSB sites and regulates HR in cells. • Single agent activity in a broad spectrum of cancer cells and enhances the sensitivity of colorectal cancer cells to RAD51 inhibitor, RI-1 and cisplatin by inducing synthetic lethality. IC_50_ = 2-25 µM	Pre-Clinical
AS4583 and RJ-LC-07-48	• Bind to the N-terminal portion of MCM2	• AS4583 inhibits the formation of the DNA replication fork by disrupting MCM complex in lung cancer cells. • AS4583 promotes ubiquitination of MCM2−7 and their degradation in lung cancer cells. • Shows single agent anticancer activity in a broad spectrum of cancer cells as well as in tyrosine kinase inhibitor (TKI)-sensitive and TKI-resistant lung cancer cells and in xenograft model. IC_50_ = 0.02-1 µM	Pre-Clinical

NR, Not Reported; DBD, DNA binding domain; NER, Nucleotide Excision Repair; GEMM, genetically-engineered mouse model; PDX, patient-derived xenograft.

## Conclusions

DSBs are the most lethal of all DNA lesions and the cadre of proteins that respond to repair of DSBs represent a diverse array of proteins and enzymes of which a small portion are in fact kinases. Combinational therapy of DSB repair inhibitors with existing DSB inducing agents has been the most effective strategy. Careful consideration of the sequence of combination drug administration and optimizing drug scheduling will likely be needed to optimize synergistic effects of combination therapy while sparing normal cells. DSB repair deficiency and mutation can increase the immunogenicity of cancers and combination of selective DSB repair inhibitors with immunotherapy could be a useful strategy in treating subsets of cancer patients. The identification of useful synthetic lethal interactions to enhance the sensitivity to widely prescribed chemotherapeutics is expected to allow more selective and efficient tumor killing with reduced toxicity. However, stratification of clinically relevant biomarkers along with extensive medicinal chemistry efforts are needed to develop novel compounds that can be exploited to discover synthetic lethal interactions with other DNA repair and DDR genes.

In the last two decades, our understanding of DSB repair pathways has improved dramatically, however, development of small molecule inhibitors targeting these repair pathways are only now being pursued in earnest and recent high-resolution protein structures of many of these putative targets can enhance these efforts. Even though, there is still an urgent need for rapid expansion of DNA repair targeted agents to move from the lab to the clinic through drug discovery and development efforts. The interdependencies between DNA repair pathways can lead to potential druggable vulnerabilities but may increase the mutagenic lesions in surviving cells and drug resistance to DSB inhibitors so a cautious approach is warranted. Thus, development of potent and selective inhibitors for each of the DSB repair proteins accompanied by robust clinical trials will have new treatment modalities for a wide range of tumors and ultimately confer benefit to human health.

## Author Contributions

All authors listed have made a substantial and direct contribution to the work. All authors have given approval to the final version of the manuscript.

## Funding

This work is supported by NIH grant R01 CA247370 (JT and NG), Wayne State University Start-up funds (NG) and the Tom and Julie Wood Family Foundation (JT).

## Conflict of Interest

KSP is a Vice-President of Research and JJT is a cofounder and CSO of NERx Biosciences.

The remaining authors declare that the research was conducted in the absence of any commercial or financial relationships that could be construed as a potential conflict of interest.

## Publisher’s Note

All claims expressed in this article are solely those of the authors and do not necessarily represent those of their affiliated organizations, or those of the publisher, the editors and the reviewers. Any product that may be evaluated in this article, or claim that may be made by its manufacturer, is not guaranteed or endorsed by the publisher.
